# Design of Tail-Clamp Peptide Nucleic Acid Tethered with Azobenzene Linker for Sequence-Specific Detection of Homopurine DNA

**DOI:** 10.3390/molecules22111840

**Published:** 2017-10-27

**Authors:** Shinjiro Sawada, Toshifumi Takao, Nobuo Kato, Kunihiro Kaihatsu

**Affiliations:** 1 Department of Organic Fine Chemicals, The Institute of Scientific and Industrial Research, Osaka University, 8-1 Mihogaoka, Ibaraki, Osaka 567-0047, Japan; shinjiro.sawada83@gmail.com (S.S.); kato-n@sanken.osaka-u.ac.jp (N.K.); 2 Laboratory of Protein Profiling and Functional Proteomics, Institute for Protein Research, Osaka University, 3-2 Yamadaoka, Suita, Osaka 565-0871, Japan; tak@protein.osaka-u.ac.jp

**Keywords:** peptide nucleic acid (PNA), tail-clamp PNA, detection of homopurine DNA, azobenzene-containing linkers

## Abstract

DNA carries genetic information in its sequence of bases. Synthetic oligonucleotides that can sequence-specifically recognize a target gene sequence are a useful tool for regulating gene expression or detecting target genes. Among the many synthetic oligonucleotides, tail-clamp peptide nucleic acid (TC-PNA) offers advantages since it has two homopyrimidine PNA strands connected via a flexible ethylene glycol-type linker that can recognize complementary homopurine sequences via Watson-Crick and Hoogsteen base pairings and form thermally-stable PNA/PNA/DNA triplex structures. Here, we synthesized a series of TC-PNAs that can possess different lengths of azobenzene-containing linkers and studied their binding behaviours to homopurine single-stranded DNA. Introduction of azobenzene at the N-terminus amine of PNA increased the thermal stability of PNA-DNA duplexes. Further extension of the homopyrimidine PNA strand at the N-terminus of PNA-AZO further increased the binding stability of the PNA/DNA/PNA triplex to the target homopurine sequence; however, it induced TC-PNA/DNA/TC-PNA complex formation. Among these TC-PNAs, 9W5H-C4-AZO consisting of nine Watson-Crick bases and five Hoogsteen bases tethered with a beta-alanine conjugated azobenzene linker gave a stable 1:1 TC-PNA/ssDNA complex and exhibited good mismatch recognition. Our design for TC-PNA-AZO can be utilized for detecting homopurine sequences in various genes.

## 1. Introduction

Sequence-specific detection of target genes using oligonucleotides is a key technology for detecting pathogens and disease-related genes. The accuracy and sensitivity of the diagnosis relies on the chemical properties of the oligonucleotides. Thus, various types of chemically modified nucleic acids have been developed to improve binding affinity and sequence specificity.

**Figure 1:**
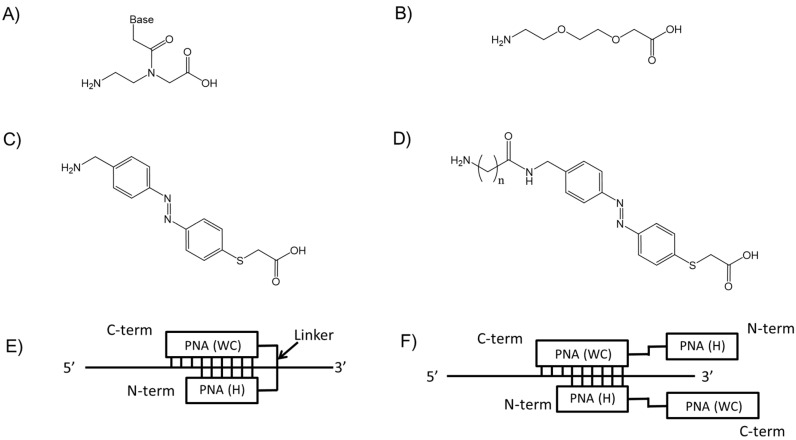
Chemical structure of (**A**) PNA monomer; (**B**) ethylene glycol-based aminoethoxyethoxy acetate linker (AEEA); (**C**) azobenzene amino acid linker (C0-AZO); and (**D**) spacer conjugated azobenzene amino acid linker (C3-, C4-, C5- and C6-AZO (*n* = 1, 2, 3 and 4)); (**E**) Schematic image of triplex formation between TC-PNA and ssDNA. The C-terminal strand (PNA (WC)) of TC-PNA was designed to form Watson-Crick base pairs and the N-terminal strand (PNA (H)) of TC-PNA was designed to form Hoogsteen base pairs with its target ssDNA; and (**F**) one possible image of a TC-PNA/TC-PNA/ssDNA trimer.

Peptide nucleic acid (PNA) is a DNA mimic in which the phosphate backbone has been replaced by a neutral amide backbone composed of *N*-(2-aminoethyl) glycine linkages (Figure 1A) [1]. The advantages of PNA are its high binding affinity [2,3,4], good mismatch discrimination [5], nuclease and protease resistance [6], and low affinity for proteins [7]. Tail-clamp PNA (TC-PNA), which is composed of two homopyrimidine PNA strands connected via a linker molecule, 2-aminoethoxy-2-ethoxy acetic acid (AEEA, Figure 1B), can form a stable complex with its target homopurine DNA sequence consisting partly of a duplex via Watson-Crick base pairing and partly of a triplex via both Watson-Crick and Hoogsteen base pairings [1,2,8,9]. Therefore, TC-PNA can form a thermally-stable PNA/DNA/PNA triplex [5,10,11]. This efficient DNA binding of TC-PNA has been used for down-regulating DNA transcription [3,12,13], DNA repair [14,15] and capturing bacterial genes [16]. We recently developed a novel type of azobenzene amino acid [17] and used it as a linker for TC-PNA to enhance the binding characteristics of PNA to a complementary RNA sequence on the influenza A virus gene [18]. 

In this study, we synthesized homopyrimidine TC-PNAs that consist of different lengths of azobenzene linker (Figure 1C,D, TC-PNA AZO) and different numbers of homopyrimidine bases strands. Their binding mode of action to complementary homopurine single stranded DNAs (ssDNAs) was assessed by gel mobility shift assay, melting temperature analysis, and electrospray ionization mass spectrometry. Optimization of both the lengths of the azobenzene linker and the homopyrimidine strands of TC-PNA efficiently provided a 1:1 complex with complementary homopurine ssDNA with high binding affinity and good sequence specificity. This result allows us to design homopyrimidine TC-PNA that recognizes homopurine DNA.

## 2. Results and Discussions

### 2.1. Design of PNAs and TC-PNAs 

We designed TC-PNA molecules composed of two PNA strands tethered with different types of spacer molecules (Table 1). TC-PNA has been demonstrated to form complex structures consisting of partially triplex and partially duplex regions with its target ssDNA by Watson-Crick and Hoogsteen base pairings [10] (Figure 1E). Since tethering two PNA strands reduces the entropic penalty paid during triplex formation by two distinct PNA molecules, TC-PNA exhibits improved binding affinity to ssDNA [19]. On the other hand, TC-PNA potentially forms a trimer complex (Figure 1F) when excess TC-PNA is added to the target ssDNA.

Recently, we reported that TC-PNA tethered with an azobenzene linker showed a nearly 10-fold improved inhibitory effect on the reverse transcription of the influenza A virus gene by MuLV reverse transcriptase [18]. These reports indicate that the linker structure of TC-PNA affects its binding characteristics with target sequences. However, homopyrimidine TC-PNAs targeting homopurine sequences may potentially form a TC-PNA/TC-PNA/DNA trimer [20]. To investigate the effect of the linker on the binding characteristics of homopyrimidine TC-PNA to homopurine ssDNA, we synthesized a series of linker molecules (Figure 1B–D) and individually introduced them as linkers for TC-PNA by Fmoc-solid phase synthesis (Table 1). The homopyrimidine PNA strand contains a complementary sequence to a target ssDNA (DNA**1**), while the linker unit contains different number of a flexible ethylene glycol based linkers (AEEA) or different lengths of rigid azobenzene linkers. PNA**1** and **2** were synthesized to confirm the effect of azobenzene on its binding characteristics to the complementary ssDNA (Table 1). PNA**3**–**5** that possess different numbers of flexible AEEA linkers were synthesized to study the effect of linker length on the binding properties of TC-PNAs (Table 1). On the other hand, PNA**6**–**10** that possess different lengths of azobenzene linkers were synthesized to study how azobenzene linkers affect the binding properties of TC-PNAs (Table 1). PNA**11**–**17** were synthesized to study the effect of the Hoogsteen strand of TC-PNA on their binding characteristics to ssDNA (Table 1). All PNAs have a lysine molecule at the C-terminus to increase water solubility. Three consecutive lysine residues at the N-terminal of the TC-PNAs were used to enhance the binding affinity of the Hoogsteen strand to the target DNA [21].

### 2.2. Recognition of Single Strand DNA by PNAs and TC-PNAs

We studied the effect of the linker structures of TC-PNA on binding efficiency to Cy-3 labelled ssDNA (DNA**1**, Cy3 and linker structure are shown in Figure S1) using gel mobility shift assays. Since Hoogseen base pairs can be formed under acidic conditions [22], we incubated TC-PNA and Cy3-ssDNA in 10 mM phosphate buffer (pH 6.0) containing 1 mM EDTA. The relative intensities of shifted bands upon non-denaturing poly-acrylamide gel electrophoresis is a measure of the binding efficiency of TC-PNA to the target ssDNA, while the mobility of each shifted band indicates the composition of the TC-PNA and ssDNA complex.

**Figure 2:**
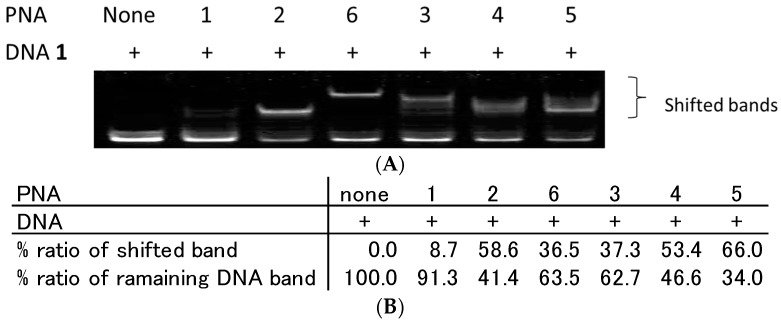
Analysis of DNA/PNA complexes by gel mobility shift assay. (**A**) All lanes contain Cy3-labelled ssDNA (DNA**1**) at 100 nM. Each lane from the left side contains no PNA, 100 nM of PNA**1**, **2**, **6**, **3**, **4**, and **5**, respectively. Each PNA and DNA**1** were incubated in 10 mM sodium phosphate buffer and 1 mM EDTA (pH 6.0) at 25 °C for 10 min. Gel mobility shift assays of PNA/DNA complexes were performed using 15% non-denaturing polyacrylamide gels; and (**B**) quantitative band-intensity analysis of DNA and DNA/PNA complexes in Figure 2.

First, we incubated PNA**1**–**6** and DNA**1** in a 1:1 molar ratio according to manufacture provided molar extinction coefficients and analysed their binding characteristics using the gel mobility shift assay. The reaction mixture was added to 30% glycerol and carefully injected in each lane of polyacrylamide gel to avoid the pH change by gel running buffer and immediately subjected to gel mobility shift assay. As a result, single strand PNA (PNA**1**) binding to DNA**1** was observed to be very limited (Figure 2A). However, PNA**2**, which has C0-AZO at the N-terminus, produced a single distinctly shifted band. This improved binding of PNA**2** can be explained by the stacking interaction of AZO with its neighbouring base pairs that increases the duplex stability (Table 2).

PNA**3**, **4**, and **5** are TC-PNAs that contain (AEEA)_1_, (AEEA)_2_, and (AEEA)_3_ as the linker molecule, respectively. Based on the amount of unbound DNA**1** shown in Figure 2A,B, PNA**5** exhibited the highest binding affinity to DNA**1** among them (shifted band; 66.0%). Corresponding to our results, Kuhn et al. reported that TC-PNA containing three consecutive AEEA linkers gives the most preferable PNA/DNA/PNA triplex with complementary ssDNA [10]. Additionally, PNA**3**–**5** afforded multiple bands with DNA**1**. A similar phenomenon has also been reported in the case of bisPNA by Hansen et al. and those multiple bands were identified as a mixture of a bisPNA/DNA complex and bisPNA/bisPNA/DNA complexes [19]. PNA**6** tethered with AZO linker showed an apparent single shifted band. The rigidity and hydrophobicity of AZO linker only allowed a preferable complex formation. On the other hand, PNA**3**–**5** showed smear shifted bands (Figure 2A). This is probably due to the flexible and water-soluble feature of AEEA linker and, thus, allowed different types of complex formation. PNA**3** and **6** that possess shorter linkers gave weaker shifted band (Figure 2B, PNA**3**; 37.3%, PNA**6**; 36.5%) than PNA**4** and **5** that possess longer AEEA linkers (Figure 2B, PNA**4**; 53.4%, PNA**5**; 66.0%). From these results, the linker length for TC-PNA could be important for complex formation with the target ssDNA.

**Figure 3:**
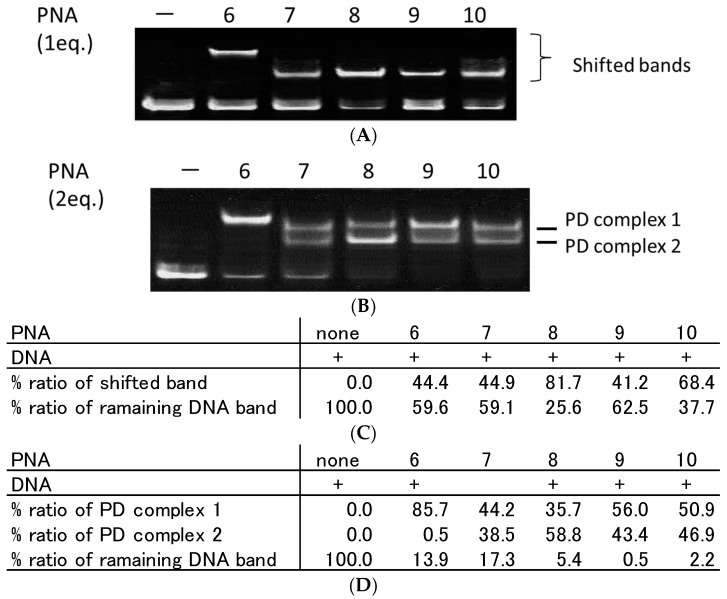
Effect of azobenzene linker length on complex formation between tail-clamp PNA (TC-PNA) and ssDNA. Each lane contains 100 nM of ssDNA. (**A**) Each lane from the left side contains no PNA, 100 nM of PNA**6**, **7**, **8**, **9**, and **10**; (**B**) Each lane from the left side contains no PNA, 200 nM of PNA**6**, **7**, **8**, **9**, and **10**, which contain C0-AZO, C3-AZO, C4-AZO, C5-AZO, and C6-AZO as linkers, respectively. Each TC-PNA and ssDNA were incubated in 10 mM sodium phosphate buffer and 1 mM EDTA (pH 6.0) at 25 °C for 10 min. Gel mobility shift assays of TC-PNA/DNA complexes were performed using 15% non-denaturing polyacrylamide gels; (**C**) quantitative band-intensity analysis of DNA and DNA/PNA complexes in Figure 3A; and (**D**) quantitative band-intensity analysis of DNA and DNA/PNA complexes in Figure 3B.

Second, we prepared azobenzene-tethered TC-PNA (PNA**6**–**10**) that contained different lengths of azobenzene linker molecules (C0-, C3-, C4-, C5-, and C6-AZO, Figure 1C,D) in between two PNA strands (Table 1). PNA**6**–**10** and DNA**1** were incubated in a 1:1 molar-ratio for 10 min at room temperature and the mixtures were assessed using a gel mobility shift assay. PNA**6**, **7**, **8**, **9**, and **10** provided a single shifted band and their relative band intensities were 44.4%, 44.9%, 81.7%, 41.2%, and 68.4%, respectively (Figure 3A,C). It is noteworthy that the mobility of PNA**6** and DNA**1** was apparently less than the others (Figure 3A). To study the stoichiometry of TC-PNA and DNA**1** in each complex, we added two equivalents of PNA**6**–**10** to DNA**1** and analysed them in the same way. Interestingly, PNA**6** gave the same single shifted band (85.7%) even with excess amounts of PNA (Figure 3B), while PNA**7**–**10** gave secondary shifted bands at lower mobility positions (Figure 3B, PD complex **1**) in addition to higher mobility positions (Figure 3B, PD complex **2**). According to our quantitative band-intensity analysis in Figure 3B,D, azobenzene linker molecules for TC-PNA affected their DNA binding properties. Further, PNA**8** that possesses C4-AZO linker showed the most efficient binding property to give PD complex **2** among PNA**6**–**10** (Figure 3C; 81.7% at one equivalent of PNA**8**, Figure 3D; 58.8% at two equivalents of PNA**8**). 

### 2.3. Effect of Homopyrimidine Bases of Azobenzene-Tethered PNAs on Complex Formation with Single Stranded DNA

**Figure 4:**
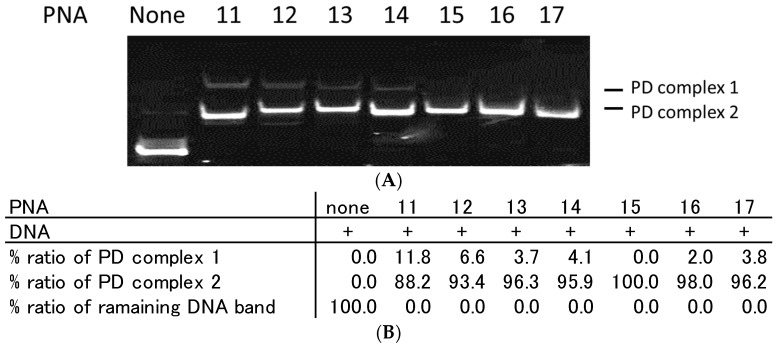
Effect of homopyrimidine bases of bisPNA and TC-PNA on complex formation with ssDNA. Each lane contains 100 nM of DNA**1**. (**A**) Each lane from the left side contains no PNA, 200 nM of PNA**11**, PNA**12**, PNA**13**, PNA**14**, PNA**15**, PNA**16**, and PNA**17**, which contain nine, eight, seven, six, five, four, and three homopyrimidine bases in the N-terminal strand of PNA, respectively. Each DNA and PNA were incubated in 10 mM sodium phosphate buffer (pH 6.0) at 25 °C for 10 min. Gel mobility shift assays of PNA/DNA complexes were performed using 15% non-denaturing polyacrylamide gels; (**B**) Quantitative band-intensity analysis of DNA and DNA/PNA complexes in Figure 4.

Azobenzene-tethered homopyrimidine TC-PNA may potentially form TC-PNA/DNA/TC-PNA trimers. Therefore, we altered the number of homopyrimidine bases of TC-PNA-C4-AZO and examined their binding properties with homopurine ssDNA by gel mobility shift assay. When two equivalents of PNA**11**–**14** were incubated with DNA**1**, there were two distinct shifted bands (Figure 4).

The lesser mobility band intensity was increased in a Hoogsteen base number dependent manner. On the other hand, PNA**15**–**17**, which have fewer Hoogsteen bases, did not afford the lesser mobility bands (Figure 4A,B). From these data, we conclude that TC-PNA–C4-AZO containing longer homopyrimidine bases tends to form a different type of complex with DNA**1**. To understand the components of the lesser mobility band, we incubated PNA**11** and DNA**1** at different pH conditions. As a result, the band intensity of PD complex **1** was slightly reduced in a pH-dependent manner, but not fully diminished (Figure S2). This indicated that the composition of the less mobile band was formed not only by Hoogsteen base pairing, but also by Watson-Crick base pairing. 

We also studied the complex formation behaviors of PNA**15** with a matched DNA (DNA**1**) and a mismatched DNA by gel mobility shift assay. As a result, PNA**15** did not form PD complex **1** with DNA**1** even in the presence of a five-fold excess of PNA**15** (Figure S3). This indicated that a stable PNA**15**/DNA**1** triplex formation prevents the binding of second PNA**15** to the complex. Further, PNA**15** did not form a complex with the mismatched DNA**3** even in the presence of a three-fold excess amounts of PNA**15** (Figure S4). However, the band intensities of DNA**3** were decreased in a PNA**15** concentration-dependent manner. PNA**15** might affect the mobility of Cy3-labelled-DNA**3** in the gel and the fluorescence intensity of Cy3-DNA smear bands could be quenched by gel components.

### 2.4. Analysis of TC-PNA and DNA Complexes Using Nano ESI-MS

**Figure 5:**
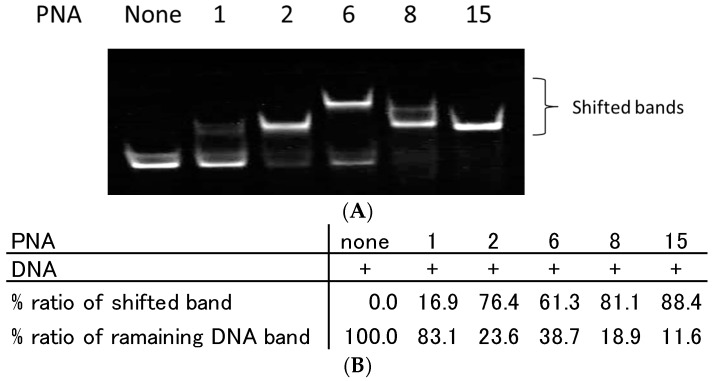
Effect of azobenzene linker length and the number of homopyrimidine bases of PNA on complex formation with complementary ssDNA. (**A**) Each lane contains 100 nM of DNA**1**. Each lane from the left side contains no PNA, 200 nM of PNA**1**, PNA**2**, PNA**6**, PNA**8**, and PNA**15**. Each DNA and PNA were incubated in 10 mM sodium phosphate buffer (pH 6.0) at 25 °C for 10 min. Gel mobility shift assays of PNA/DNA complexes were performed using 15% non-denaturing polyacrylamide gels. (**B**) Quantitative band-intensity analysis of DNA and DNA/PNA complexes in Figure 5.

To analyse the composition of TC-PNA and ssDNA complexes, we employed PNA**6**, **8** and **15**, since they exhibited a different gel mobility shift pattern as seen in Figure 3A,B and Figure 4. Indeed, the addition of two equivalents of PNA**6**, **8**, or **15** to DNA**1** produced single bands with a lesser mobility band (61.3%), two shifted bands (81.8% in total), or a greater mobility band (88.4%), respectively (Figure 5A,B).

The composition of the PNA**6**/DNA**1** complex was analysed using nano ESI-MS. As a result, we observed a set of multiply charged ion peaks at 1845.8 (+10; calcd. 1845.7), 2049.4 (+9; calcd. 2050.6) and 2308.8 (+8; calcd. 2308.7 that agree with the theoretical molecular mass (18,447.9 Da) of a PNA**6**/PNA**6**/DNA**1**/Na^+^ complex (Figure S5A, Table S1; Supplemental Data). This indicated that PNA**6** initially formed Watson-Crick base pairing with DNA**1**; however, the length of the C0-AZO linker was insufficient to facilitate Hoogsteen base pairing. Thus, a second PNA**6** was bound to the 1:1 PNA**6**/DNA**1** complex and afforded a PNA**6**/DNA**1**/PNA**6** complex. 

In the case of PNA**8**, we observed a set of multiply charged ion peaks at 1899.7 (+7; calcd. 1900.6) that agree with the theoretical molecular mass (13,298.3 Da) of a 1:1 PNA**8**/DNA**1**/Na^+^ complex (Figure S5B, Table S2; Supplemental Data). We also observed another set of multiply-charged ion peaks at 1859.9 (+10; calcd. 1859.9), 2065.5 (+9; calcd. 2066.4) and 2323.5 (+8; calcd. 2324.6) that agree with the theoretical molecular mass (18,590.1 Da) of a PNA**8**/PNA**8**/DNA**1**/Na^+^ complex (Figure S5B, Table S2; Supplemental data). Taken together with our previous gel mobility shift assay data, the linker substitution of TC-PNA from C0-AZO to C4-AZO facilitates a 1:1 TC-PNA/ssDNA complex formation. 

PNA**15**, which possesses a shorter homopyrimidine strand, produced a set of multiply-charged ion peaks at 1565.5 (+8; calcd. 1565.0), 1789.1 (+7; calcd. 1788.2), and 2086.9 (+6; calcd. 2086.4 that agree with the theoretical molecular mass (12,513.6 Da) of a PNA**15**/DNA**1**/Na^+^ (Figure S5C, Table S3; Supplemental data). Side peaks at 1546.8 (+8; calcd. 1544.4), 1767.6 (+7; calcd. 1764.9), and 2062.3 (+6; calcd. 2058.9), which agree with the theoretical molecular mass (12,348 Da) of the 1:1 complex of PNA**15**/DNA**1**/Na^+^ lacking a lysine residue, were also observed. 

From these results, homopyrimidine TC-PNA-AZO potentially forms 2:1 complexes with homopurine ssDNA. This was also confirmed in the case of ssPNA (Figure S6A: PNA**1**, Figure S6B: PNA**2**, Tables S4 and S5; Supplemental Data). However, the 2:1 complex formation can be prevented by introducing a modest length of azobenzene linker into TC-PNA and reducing the number of homopyrimidine strands that can form Hoogsteen base pairs.

### 2.5. Thermal Stability of TC-PNA and ssDNA Complex

The thermal stability of PNA and DNA gives us some insights into their binding modes of action. Thus, we measured the melting temperature of our representative PNAs and DNA**1**. As a result, PNA**2** showed approximately 5 °C higher Tm compared to PNA**1**/DNA**1**, probably due to the stacking interaction of azobenzene to the neighbouring bases (Table 2). PNA**5**, which has three consecutive AEEA linkers and five extended homopyrimidine bases, exhibited 18.3 °C higher *T*_m_ compared to PNA**1** (Table 2). The three extended AEEA linkers and five homopyrimidine PNA bases contributed to stabilize the TC-PNA/DNA duplex. PNA**6** and PNA**9**, which possess different lengths of azobenzene linkers, both exhibited approximately 11–13 °C higher TC-PNA/DNA duplex stability than PNA**1**, which was approximately 5–7 °C lower than that of PNA**5** (Table 2). Although the azobenzene linker by itself tends to increase the PNA/DNA duplex stability by stacking interactions, the extended homopyrimidine PNA bases might have limited the stacking interaction by forming PNA/DNA base pairing.

To study the effect of homopyrimidine bases of TC-PNA-C4-AZO on its interaction with ssDNA, we analysed the T_m_ of PNA**11**(9W9H-AZO), **13**(9W7H-AZO), **15**(9W5H-AZO) and **17**(9W3H-AZO) with DNA**1**. As a result, the Tm of PNA**11**, **13**, **15**, and **17** with DNA**1** were 19.9 °C, 14.5 °C, and 8.3 °C higher than that of PNA**17**/DNA**1**, respectively. This result indicated that the extended homopyrimidine bases contribute to thermally stabilize the duplex in a sequence and length dependent manner. However, the extended homopyrimidine bases may induce a second PNA binding as shown in Figure 4. Optimizing the design of PNA is important not only for increasing its binding affinity, but also eliminating non-specific interactions with the target ssDNA.

### 2.6. Strand Displacement of dsDNA by TC-PNA-AZO 9W5H-C4

**Figure 6:**
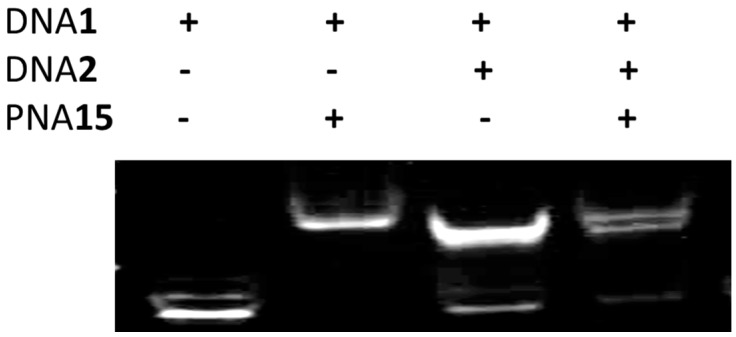
Strand displacement of dsDNA by TC-PNA-C4-AZO. Each lane contains oligonucleotides as indicated above. The concentrations of DNA**1** and DNA**2** were 100 nM, while PNA**15** was 200 nM. Each DNA and PNA were incubated in 10 mM sodium phosphate buffer (pH 6.0) at 25 °C for 10 min. Gel mobility shift assays of PNA/DNA complexes were performed using 15% non-denaturing polyacrylamide gels.

To determine if TC-PNA-C4-AZO can recognize a target sequence within duplex DNA, we prepared dsDNA by annealing 5′-Cy3-labelled target ssDNA (DNA**1**) and a complementary ssDNA (DNA**2**, Table 1). DNA**1**, DNA**2**, and the duplex DNA were individually incubated with PNA**15** for 10 min at room temperature and subjected to gel mobility shift assays. PNA**15** bound DNA**1** and produced a single shifted band (Figure 6). When PNA**15** was incubated with dsDNA, the duplex band intensity was reduced and a new, shifted band appeared on the gel (Figure 6).

The mobility of the shifted band was identical to the band observed in the lane containing PNA**15** and DNA**1**. This indicated that PNA**15** can recognize the target sequence in dsDNA by forming a complex with DNA**1** and eliminating DNA**2** from the duplex. Interestingly, PNA**15** did not induce multi-complex formation, even when five equivalents of PNA**15** were added to DNA**1** (Figure S3). Further, PNA**15** also recognized mismatched bases in DNA**3** (Figure S4).

## 3. Materials and Methods

### 3.1. Chemicals

Fmoc/Bhoc-protected PNA monomers were purchased from Panagene (Daejeon, Korea). Fmoc-Lys(Boc)-OH, poly-ethylene linker and TGR-resin were purchased from Merck Millipore (Tokyo, Japan). The coupling activators and HATU were purchased from Watanabe Chemicals (Hiroshima, Japan). DNA (salt free) was purchased from Sigma-Genosys (Ishikari, Japan). Other chemicals were purchased from Wako Pure Chemical (Osaka, Japan), Sigma-Aldrich (Tokyo, Japan) and Tokyo Chemical Industry (Tokyo, Japan). Reagents and solvents were used without further purification unless otherwise noted.

### 3.2. Preparation of Fmoc-Lys-(Boc)-OH Loaded Resin

The Novasyn TGR resin (200 mg, 0.24 mmol/g) was swollen in 5 mL DMF for 30 min prior to the synthesis. To activate the carboxyl groups of coupling monomers, 200 μL of base solution (0.3 M 2,6-lutidine, 0.2 M diisopropyl ethylamine and 0.33 M thiourea solution), and 0.5 M HATU were added to a mixture of Fmoc/Boc-protected lysine (Fmoc-Lys(Boc)-OH, 22.5 mg, 48 μmol) and Boc/Cbz-protected lysine (Boc-Lys(Cbz)-OH, 72.3 mg, 190 μmol) in 200 μL of DMF. For the coupling reaction, the mixture was added to the resin and incubated at ambient temperature for 60 min. The resin was then removed from the reaction solution and washed with DMF (5 × 5 mL). For capping of non-reacted amine groups, the resin was treated with 1.5 mL of capping solution (2,6-lutidine:Ac_2_O:pyridine = 6:5:89, *v*/*v*) for 5 min and washed with DMF (10 × 5 mL). For deprotection of Fmoc-groups, the resin was treated with 1 mL of deblock solution (40% (*v*/*v*) piperidine in DMF) for 5 min and washed with DMF (10 × 5 mL).

### 3.3. PNA Synthesis

Automated linear solid phase synthesis of PNA was performed using an Intavis ResPep parallel synthesizer equipped with micro scale columns (Köln, Germany). The lysine-loaded resin (200 mg, 16 μmol) was swollen in 5 mL DMF for 30 min and 20 mg of the resin was transferred to each column in the synthesizer. After removal of DMF, Fmoc-protecting groups of the lysine-loaded resin were removed from the resin by a 10-min incubation in 100 μL of deblock solution; the resin was subsequently washed with 100 μL of DMF 10 times. The concentration of each monomer (Fmoc-PNA monomers, Fmoc-AEEA-OH, Fmoc-AZO-OH [17] and Fmoc-Lys(Boc)-OH) was adjusted to 0.3 M in DMF solution. The activator solution contained 0.5 M HATU in DMF. The base solution contained 0.3 M 2,6-lutidine, 0.2 M diisopropyl ethylamine and 0.33 M thiourea in 5% NMM in pyridine solution (*v*/*v*). The deblock solution contained 40% pyperidine in DMF solution.

For the coupling reaction, 17.5 μL of monomer solution, 17.0 μL of activation solution and 8.50 μL of base solution were combined in a vessel and incubated for 2 min at ambient temperature. The coupling solution was then transferred to 20 mg of the resin and then incubated for 100 min at ambient temperature. After eluting the coupling solution from the resin by filtration, the resin was washed with 100 μL of DMF 10 times. This coupling procedure was repeated twice for each monomer elongation reaction. The resin was then incubated with 100 μL of capping solution for 10 min to protect the non-elongated amino groups with acetyl groups and subsequently washed with 100 μL of DMF 10 times. The N-terminus Fmoc-group was then removed by incubating the resin with 100 μL of deblock solution for 10 min and subsequently washing with 100 μL of DMF 10 times. The coupling step of the next monomer and capping steps were repeated as described above until the desired PNA molecule was synthesized. Before the cleavage of PNA molecules from the resin, the resin was washed with 100 μL of DMF five times followed by washing with 100 μL of dichloromethane five times. After drying the resin, 1 mL of TFA/m-cresol (9:1, *v*/*v*) was added and incubated for 12 h. The resin was then filtered and the flow-through containing the cleaved PNA was transferred to a new tube. The PNA solution was added to 15 mL of ice-cold diethyl ether and the precipitate was collected by centrifugation at 4400 rpm for 4 min. The supernatant was transferred to another tube and discarded. The residue was dried under ambient atmosphere and then dissolved in 100 μL of distilled water.

### 3.4. PNA Purification and Analysis

All PNAs were purified by reverse-phase HPLC using a JASCO PU-2086 pump system (Tokyo, Japan) with a JASCO UV-2075 detector and a GL Science (Tokyo, Japan) Inertsil (150 mm × 4.6 mm, 5 μm) C-18 column for analytical runs or a GL Science Inertsil (20 mm × 250 mm, 3 μm) C-18 column for semi-preparative runs. Eluting solvents (analytical: A (0.1% TFA in water) and B (0.1% TFA in acetonitrile); semi preparative: A (0.1% TFA in water) and B (0.1% TFA in acetonitrile)) were used in a linear gradient at a flow rate of 1 mL/min for analytical and 5 mL/min for semi-preparative HPLC. The gradient for analytical runs was 0 → 50% B in 30 min and the gradient for semi-preparative runs was 0% B for 10 min, 0 → 5% B in 10 min, 5% B for 10 min, 5 → 10% B in 10 min, 10% B for 10 min, 10 → 20% B in 120 min, and 20 → 50% B in 10 min. Detection was performed using a UV-VIS detector at 260 nm. PNA molecular weights were analysed using an Ultraflextreme MALDI TOF Mass Spectrometer (Bruker Daltonics, Yokohama, Japan) (Figures S7–S23). The optical densities of PNA and DNA were measured at 260 nm with a UV1700 spectrometer (Shimadzu, Kyoto, Japan) using quartz cuvettes (4 × 10 mm). The extinction coefficient of PNA was calculated from the molar extinction coefficient obtained from https://secure.eurogentec.com/EGT/files/FileBrowse/Brochures/Oligonucleotides/PNA-guide.pdf. Measurements of absorption at 260 nm were carried out in a buffer solution (10 mM NaH_2_PO_4_, pH 7.0) at ambient temperature.

### 3.5. Gel Mobility Shift Analysis of PNA/DNA Complexes

PNAs were preheated at 95 °C for 5 min to prevent aggregation, then gradually cooled to 25 °C before being added to the DNA solution. DNA concentrations were quantified by absorbance at 260 nm using a molar extinction coefficient provide by manufacturer. PNA/DNA hybridization assays were conducted using 100 nM Cy3-labeled ssDNA with 1–2 equivalents of ssPNA, bisPNA, or TC-PNA in 10 mM sodium phosphate and 1 mM EDTA at pH 6.0 for 10 min at 25 °C. The reaction mixture for each condition was mixed with 0.2 volumes of a solution containing 30% glycerol, 0.025% bromophenol blue, and 0.025% xylencyanol (Sigma) and subjected to electrophoresis at 20 mA for 30 min on a 15% non-denaturing polyacrylamide gel using 1× TBE as a running buffer (89 mM Tris base, 89 mM borate, 2 mM EDTA, pH 8.1) at 4 °C in the dark. The gel images were created by use of a CCD digital image stock system, FAS-III (Toyobo, Osaka, Japan).

### 3.6. Analysis of TC-PNA/DNA Complexes Using Nano ESI-MS

The TC-PNA/DNA complexes were incubated under the conditions described above. The mixture was diluted and analysed by nano electrospray ionization mass spectrometry (nano ESI-MS) in 100 mM AcONH_4_ aqueous solution. The experiments were performed on a Q-TOF II mass spectrometer (Micromass, Manchester, UK) in positive ion mode. To minimize complex dissociation, the capillary and cone voltages were optimized to 1.2 kV and 30 V, respectively. The nitrogen counter gas temperature was adjusted to 30 °C or 80 °C. Ar gas was introduced into a collision cell potentiated at 10 V, while the sample solution was introduced into a glass capillary (Proxeon, Thermo Fisher Scientific Inc., Waltham, MA, USA). Mass calibration was performed using cluster ions derived from NaI.

### 3.7. Thermal Melting Analaysis of PNA/DNA and TC-PNA/DNA

PNAs were preheated at 95 °C for 5 min to prevent aggregation, then gradually cooled to 25 °C before being added to the DNA solution. Melting profiles of PNA complexed with ssDNA were analysed on a UV1700 spectrophotometer (Shimadzu) using a microcell (eight cell, 1 mm) at 260 nm. PNAs and DNA1 were suspended in Na_2_HPO_4_ buffer (10 mM, pH 6.0) at 1 μM each. The temperature was ramped down from 95 °C to 10 °C at a rate of −1 °C/min.

## 4. Conclusions

In conclusion, homopyrimidine TC-PNA consisting of a C4-AZO linker, nine homopyrimidine bases in the N-terminal strand, and five homopyrimidine bases in the C-terminal strand can be used for detecting homopurine DNA target sequences with good binding affinity and sequence specificity. Our study into the design of TC-PNA should expand potential target sites and eliminate non-specific binding to other homopurine target sequences within a given gene.

## Figures and Tables

**Table 1:** PNAs, TC-PNAs, and ssDNAs used in this study.

Entry	Name	PNA Sequence (N to C)/DNA Sequence (5′ to 3′)	Mass	
			Calcd.	Found
PNA**1**	12W	TCTCCCTTCTTT-Lys	3266.44	3267.81
PNA**2**	12W-C0-AZO	AZO-TCTCCCTTCTTT-Lys	3549.80	3550.89
PNA**3**	12W5H-(AEEA)	H-(Lys)_3_-CCTCT-(AEEA)-TCTCCCTTCTTT-Lys	5082.51	5083.10
PNA**4**	12W5H-(AEEA)_2_	H-(Lys)_3_-CCTCT-(AEEA)_2_- TCTCCCTTCTTT-Lys	5227.70	5228.86
PNA**5**	12W5H-(AEEA)_3_	H-(Lys)_3_-CCTCT-(AEEA)_3_-TCTCCCTTCTTT-Lys	5372.80	5373.63
PNA**6**	12W5H C0-AZO	H-(Lys)_3_-CCTCT-AZO-TCTCCCTTCTTT-Lys	5220.74	5221.94
PNA**7**	12W5H C3-AZO	H-(Lys)_3_-CCTCT-C3-AZO-TCTCCCTTCTTT-Lys	5277.81	5278.77
PNA**8**	12W5H C4-AZO	H-(Lys)_3_-CCTCT-C4-AZO-TCTCCCTTCTTT-Lys	5291.82	5292.61
PNA**9**	12W5H C5-AZO	H-(Lys)_3_-CCTCT-C5-AZO-TCTCCCTTCTTT-Lys	5305.86	5306.59
PNA**10**	12W5H C6-AZO	H-(Lys)_3_-CCTCT-C6-AZO-TCTCCCTTCTTT-Lys	5319.89	5320.01
PNA**11**	9W9H C4-AZO	H-(Lys)_3_-CTTCCCTCT-C4-AZO-TCTCCCTTC-Lys	5528.17	5529.19
PNA**12**	9W8H C4-AZO	H-(Lys)_3_-TTCCCTCT-C4-AZO-TCTCCCTTC-Lys	5276.87	5277.21
PNA**13**	9W7H C4-AZO	H-(Lys)_3_-TCCCTCT-C4-AZO-TCTCCCTTC-Lys	5010.62	5011.17
PNA**14**	9W6H C4-AZO	H-(Lys)_3_-CCCTCT-C4-AZO-TCTCCCTTC-Lys	4744.37	4745.22
PNA**15**	9W5H C4-AZO	H-(Lys)_3_-CCTCT-C4-AZO-TCTCCCTTC-Lys	4493.07	4494.28
PNA**16**	9W4H C4-AZO	H-(Lys)_3_-CTCT-C4-AZO-TCTCCCTTC-Lys	4241.77	4242.89
PNA**17**	9W3H C4-AZO	H-(Lys)_3_-TCT-C4-AZO-TCTCCCTTC-Lys	3990.47	3991.77
DNA**1**		Cy3-CATCATCAAAGAAGGGAGATGGTG	7982.50	
DNA**2**		CCATCTCCCTTCTTTGATGATG	6627.40	
DNA**3**		Cy3-CATCATCAAATAAGGTAGATGGTG	7932.40	

AEEA: 2-aminoethoxy-2-ethoxy acetic acid, AZO: Thioazobenzene, Lys: Lysine. Underline: Mismatch bases.

**Table 2:** Melting temperatures between PNAs or TC-PNAs and DNA**1**.

PNA	Name	T_m_	ΔT_m_ *	ΔT_m_ **
**1**	12W	68.0	-	
**2**	12W-C0-AZO	72.9	4.9	
**6**	12W5H-C0-AZO	81.5	13.5	
**9**	12W5H-C4-AZO	79.3	11.3	
**5**	12W5H-(AEEA)_3_	86.3	18.3	
**11**	9W9H-C4-AZO	79.6		19.9
**13**	9W7H-C4-AZO	74.3		14.6
**15**	9W5H-C4-AZO	68.0		8.3
**17**	9W3H-C4-AZO	59.7		-

* melting temperature difference relative to PNA**1**(12W)/DNA**1**; ** melting temperature difference relative to PNA**17**(9W3H-C4-AZO)/DNA**1.**

## Supplementary Materials

The following are available online.

## Abbreviations

The following abbreviations are used in this manuscript:

AEEA	Aminoethoxy-2-ethoxy acetic acid
BCIP	5-Bromo-4-chloro-3′-indolylphosphatase p-toluidine salt
Bhoc	Benzhydryloxycarbonyl
Boc	Tert-butyloxycarbonyl
cDNA	Complementary DNA
DMF	*N*,*N*-Dimethylformamide
Fmoc	9-Fluorenylmethoxycarbonyl
HBTU	2-(1*H*-Benzotriazole-1-yl)-1,1,3,3-tetramethyluronium hexafluorophosphate
HOBt	*N*-Hydroxybenzotriazole
NMM	*N*-Methylmorpholine
TFA	Trifluoroacetic acid
PNA	peptide nucleic acid

## References

[1] Nielsen P.G., Egholm M., Berg R.H., Buchardt O. (1991). Sequence-selective recognition of DNA by strand displacement with a thymine-substituted polyamide. Science.

[2] Hanvey J.C., Peffer N.J., Bisi J.E., Thomson S.A., Cadilla R., Josey J.A., Ricca D.J., Hassman C.F., Bonham M.A., Au K.G. (1992). Antisense and antigene properties of peptide nucleic acids. Science.

[3] Larson H.J., Bentin T., Nielsen P.E. (1999). Antisense properties of peptide nucleic acid. Biochim. Biophys. Acta.

[4] Ray A., Norden B. (2000). Peptide nucleic acid (PNA): Its medical and biotechnical applications and promise for the future. FASEB J..

[5] Egholm M., Buchardt O., Christensen L., Behrens C., Freier S.M., Driver D.A., Berg R.H., Kim S.K., Norden B., Nielsen P.E. (1993). PNA hybridizes to complementary oligonucleotides obeying the Watson-Crick hydrogen-bonding rules. Nature.

[6] Demidov V.V., Potaman V.N., Frank-Kamenetskii M.D., Egholm M., Buchard O., Sonnichsen S.H., Nielsen P.E. (1994). Stability of peptide nucleic acids in human serum and cellular extracts. Biochem. Pharmacol..

[7] Hamilton S.E., Iyer M., Norton J.C., Corey D.R. (1996). Specific and non-specific inhibition of RNA synthesis by DNA, PNA and phosphorothioate promoter analog duplexes. Bioorg. Med. Chem. Lett..

[8] Cherny D.Y., Belotserlovski B.P., Frank-Kamenetskii M.D., Egholm M., Buchardt O., Berg R.H., Nielsen P.E. (1993). DNA unwinding upon strand-displacement binding of a thymine-substituted polyamide to double-stranded DNA. Proc. Natl. Acad. Sci. USA.

[9] Nielsen P.E. (2000). Targeting Double Stranded DNA with Peptide Nucleic Acid (PNA). Curr. Med. Chem..

[10] Kuhn H., Demidov V.V., Nielsen P.E., Frank-Kamenetskii M.D. (1999). An experimental study of mechanism and specificity of peptide nucleic acid (PNA) binding to duplex DNA. J. Mol. Biol..

[11] Kaihatsu K., Braasch D.A, Cansizoglu A., Corey D.R. (2002). Enhanced strand invasion by peptide nucleic acid-peptide conjugates. Biochemistry.

[12] Larsen H.J., Nielsen P.E. (1996). Transcription-mediated binding of peptide nucleic acid (PNA) to double-stranded DNA: Sequence-specific suicide transcription. Nucleic Acids Res..

[13] Kaihatsu K., Shah R.H., Zhao X., Corey D.R. (2003). Extending recognition by peptide nucleic acids (PNAs): Binding to duplex DNA and inhibition of transcription by tail-clamp PNA-Peptide conjugates. Biochemistry.

[14] Schleifman E.B., Bindra R., Leif J., del Campo J., Rogers F.A., Uchil P., Kutsch O., Shuults L.D., Kumar P., Greiner D.L., Glazer P.M. (2011). Targeted disruption of the CCR5 gene in human hematopoietic stem cells stimulated by peptide nucleic acids. Chem. Biol..

[15] Bahal R., McNeer N.A., Quijano E., Liu Y., Sulkowski P., Turchick A., Lu Y.C., Bhunia D.C., Manna A., Greiner D.L. (2016). In vivo correction of anaemia in β-thalassemic mice by ΥPNA-mediated gene editing with nanoparticle delivery. Nat. Commun..

[16] Smolina I., Miller N.S., Frank-Kamenetskii M. (2010). PNA-based microbial pathogen identification and resistance marker detection: An accurate, isothermal rapid assay based on genome-specific features. Artif. DNA PNA XNA.

[17] Sawada S., Kato N., Kaihatsu K. (2012). Synthesis and application of visible light sensitive azobenzene. Curr. Pharm. Biotechnol..

[18] Kaihatsu K., Sawada S., Nakamura S., Nakaya T., Yasunaga T., Kato N. (2013). Sequence-specific and visual identification of the influenza virus NS gene by azobenzene-tethered bis-peptide nucleic acid. PLoS ONE.

[19] Griffith M.C., Risen L.M., Greig M.J., Lesnik E.A., Sprankle K.G., Griffey R.H., Kiely J.S., Freier S.M. (1995). Evaluation of pyrimidine PNA binding to ssDNA targets from nonequilibrium melting experiments. J. Am. Chem. Soc..

[20] Hansen G.I., Bentin T., Larsen H.J., Nielsen P.E. (2001). Structural isomers of bis-PNA to a target in duplex DNA. J. Mol. Biol..

[21] Silvester N.C., Bushell G.R., Searlesa D.J., Brown C.L. (2007). Effect of terminal amino acids on the stability and specificity of PNA–DNA hybridisation. Org. Biomol. Chem..

[22] Amodio A., Zhao B., Porchetta A., Idili A., Castronovo M., Fan C., Ricci F. (2014). Rational design of pH-controlled DNA strand displacement. J. Am. Chem. Soc..

