# Supplementary material for: Design of Tail-Clamp Peptide Nucleic Acid Tethered with Azobenzene Linker for Sequence-Specific Detection of Homopurine DNA
Source: Molecules. 2017 Oct 27;22(11):1840. doi: 10.3390/molecules22111840 (PMC6150319; doi:10.3390/molecules22111840)
Supplement: Supplementary file 1 [file molecules-22-01840-s001.pdf]

molecules

MDPI

Article

Design of tail-clamp peptide nucleic acid tethered with azobenzene linker for sequence-specific detection of homopurine DNA

Shinjiro Sawada [1], Toshifumi Takao [2], Nobuo Kato [1] and Kunihiro Kaihatsu [1,*]

* Correspondence: kunihiro@sanken.osaka-u.ac.jp; Tel.: +81-6-6879-8472

Supporting Information

Table of Contents: S2

Supplementary Figures

Figure S1 S3
Figure S2 S4
Figure S3 S5
Figure S4 S6
Figure S5 S7
Figure S6 S8

MALDI-TOF-MS analysis data of PNA**1-17**
Figure S7–S23 S9–17

Estimated multiply charged ion peaks of PNA/DNA complexes
Table S1-5 S18–22

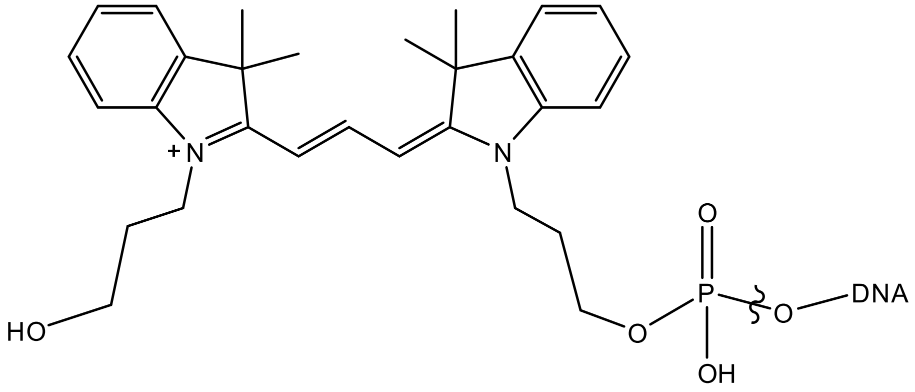

Figure S1. Chemical structure of Cy3 modified at 5′ termini of DNA**1** and DNA**3**.

A)

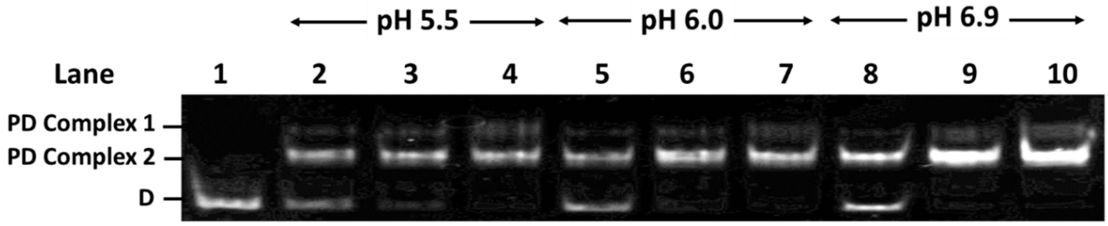

B)

PNA	1	2	3	4	5	6	7	8	9	10
DNA	+	+	+	+	+	+	+	+	+	+
% ratio of PD complex 1 band	0.00	13.37	20.07	29.11	7.75	13.60	21.43	7.28	13.63	19.42
% ratio of PD complex 2 band	0.00	62.67	72.99	69.63	63.37	82.77	78.57	70.23	83.65	78.52
% ratio of ramaining DNA band	100.00	23.96	6.94	1.25	28.88	3.64	0.00	22.50	2.72	2.05

Figure S2. Effect of pH on complex formation of PNA**11** and DNA**1** under different pH conditions. A) Each lane contains 100 nM of DNA**1**. Lane 1 contains no PNA. Lanes 2, 5, and 8 contain 1 equivalent of PNA**11**. Lanes 3, 6, and 9 contain 2 equivalents of PNA**11**. Lanes 4, 7, and 10 contain 3 equivalents of PNA**11**. Each DNA and PNA were incubated in 10 mM sodium phosphate buffer (pH6.0) at 25°C for 10 min. Gel mobility shift assays of PNA/DNA complexes were performed using 15% non-denaturing polyacrylamide gels. B) Relative band intensities of PNA/DNA complex 1 (PD complex 1), PNA/DNA complex 2 (PD complex 2), and DNA**1** in a gel mobility shift assay at different PNA molar ratios and pH conditions. B) Quantitative band-intensity analysis of DNA and DNA/PNA complexes in Fig. S2A.

A)

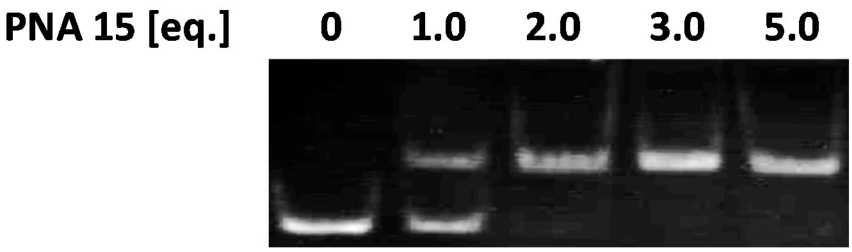

B)

PNA 15 (eq.)	0	1	2	3	5
DNA 1	+	+	+	+	+
% ratio of shifted band	0.00	39.83	76.48	80.44	75.21
% ratio of ramaining DNA band	100.00	60.17	23.52	19.56	24.79

Figure S3. Gel mobility shift analysis of complex formation between DNA**1** and PNA**15**. All lanes contain Cy3-labelled single strand DNA **1** (100 nM) and varying amounts of *trans*-AZO PNA**15**. The configuration of azobenzene was thermally isomerized to the *trans*-form by incubating at 95°C for 10 min prior to the experiment. PNA and ssDNA were incubated in 10 mM sodium phosphate buffer (pH6.0) at 25°C for 10 min. The gel mobility shift assay of PNA/ssDNA complex formation was performed using a 15% non-denaturing polyacrylamide gel. B) Quantitative band-intensity analysis of DNA and DNA/PNA complexes in Fig. S3A.

A)

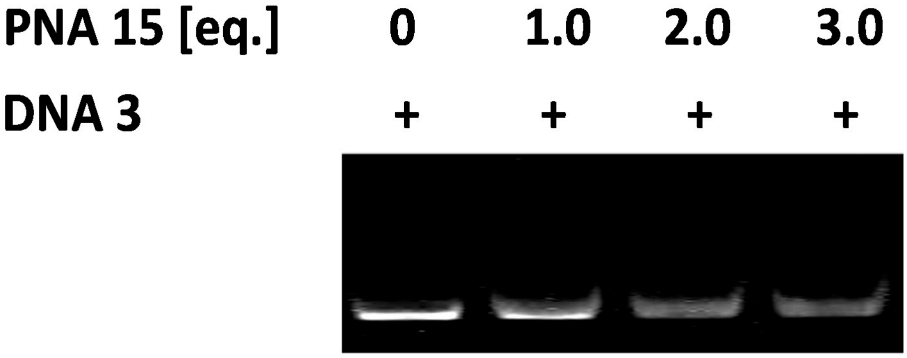

B)

PNA 15 (eq.)	0	1	2	3
% ratio of ramaining DNA band	45.651	43.893	34.13	29.48

Figure S4. Mismatch base recognition of PNA**15** on the target DNA**3**. A) Excess amounts of PNA**15** were added to each ssDNA at various concentrations, and complex formation was monitored using gel mobility shift assays. The configuration of azobenzene was thermally isomerized to the *trans*-form by incubating it at 95°C for 10 min prior to the experiment. PNA**15** and ssDNA were incubated in 10 mM sodium phosphate buffer (pH6.0) at 25°C for 10 min. The gel mobility shift assay of PNA/ssDNA complex formation was performed using a 15% non-denaturing polyacrylamide gel. B) Quantitative band-intensity analysis of DNA**3** in Fig. S4A.

A)

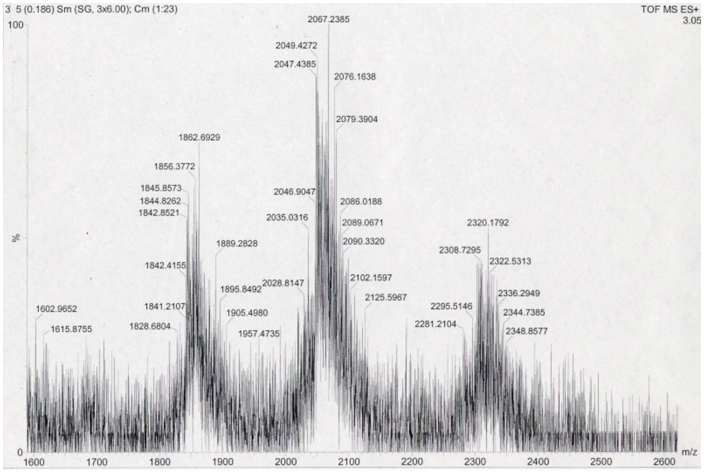

B)

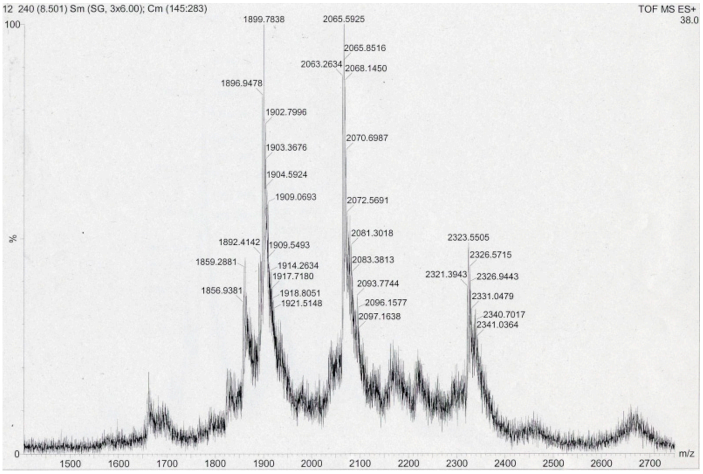

C)

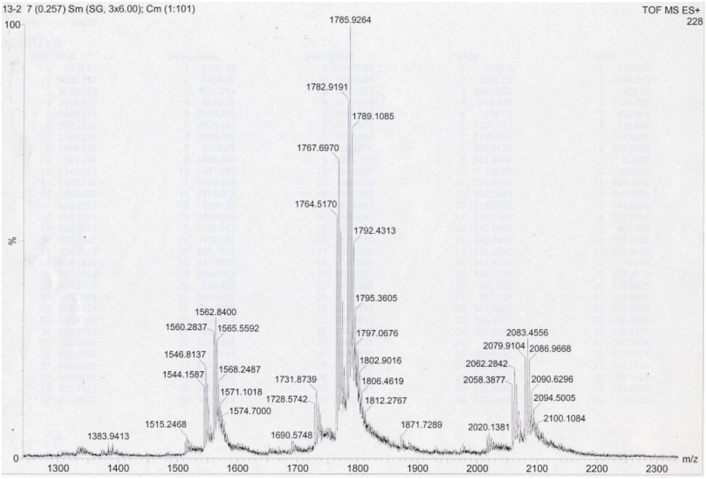

Figure S5. Stoichiometry analysis of ssDNA and TC-PNA complexes by Nano ESI-mass spectrometry. A) PNA**6** and DNA**1** were incubated at a 2:1 molar ratio (200 nM, 100 nM). B) PNA**8** and DNA**1** were incubated at a 2:1 molar ratio (200 nM, 100 nM). C) PNA**15** and DNA**1** were incubated at a 2:1 molar ratio (200 nM, 100 nM).

A)

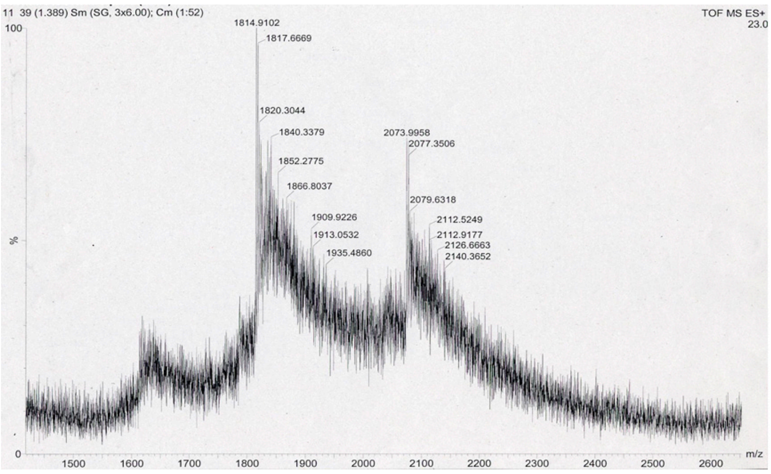

B)

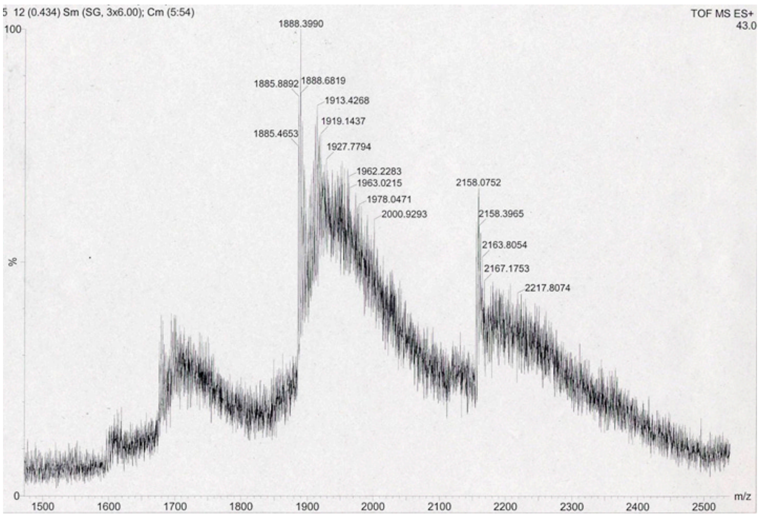

Figure S6. Stoichiometry analysis of ssDNA and PNA complexes by Nano ESI-mass spectrometry. A) PNA**1** and DNA**1** were incubated at a 2:1 molar ratio (200 nM, 100 nM). B) PNA2 and DNA1 were incubated at a 2:1 molar ratio (200 nM, 100 nM).

MALDI–TOF-MS analysis of PNAs

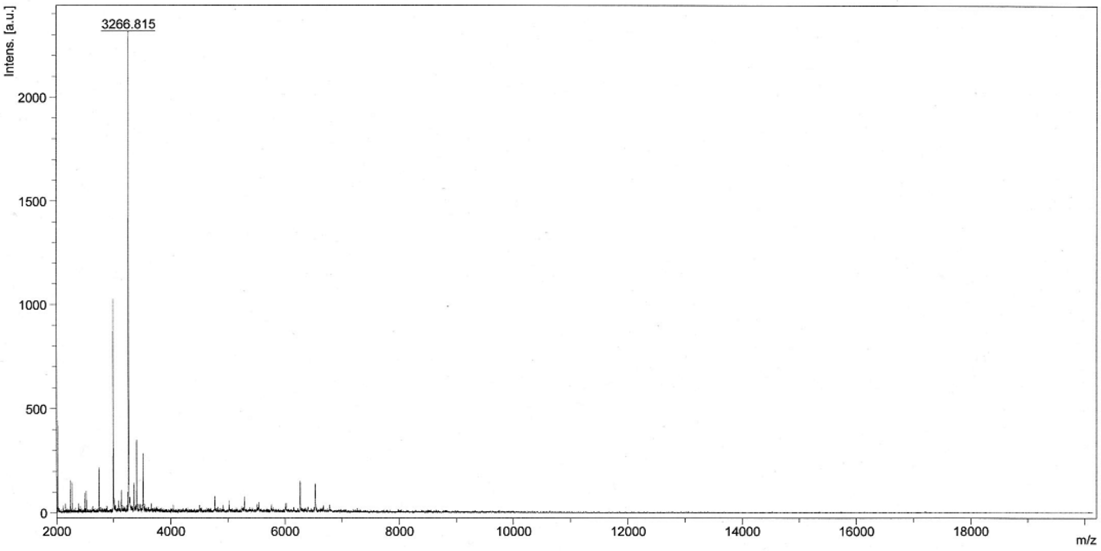

Figure S7. PNA**1**:TCTCCCTTCTTT-K.

MALDI-TOF-MS (sinapinic acid): m/z calcd. 3266.44, observed 3266.815 [M(average)] $^{+}$.

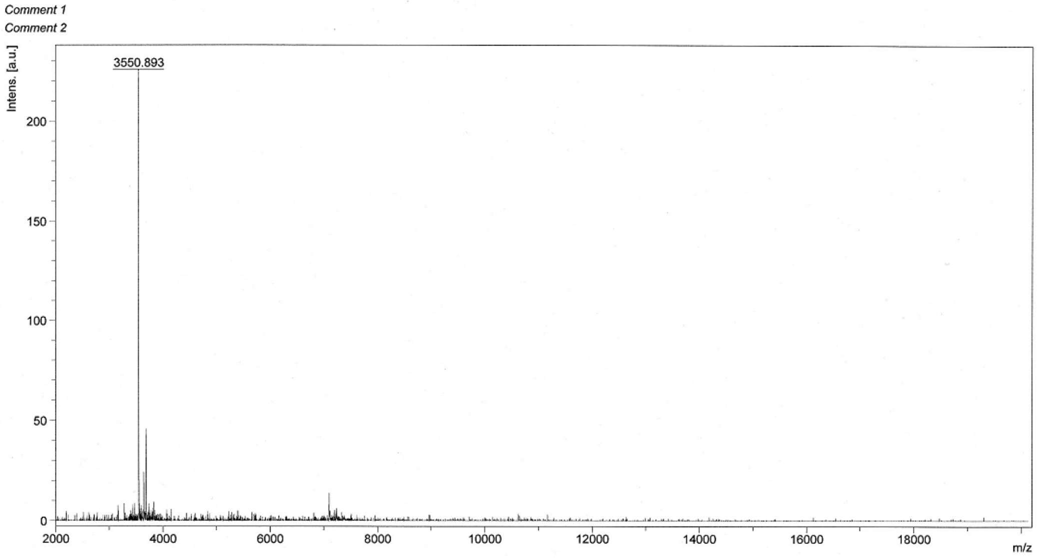

Figure S8. PNA**2**: AZO-TCTCCCTTCTTT-K.

MALDI-TOF-MS (sinapinic acid): m/z calcd. 3549.80, observed 3550.89 [M(average)]$^+$.

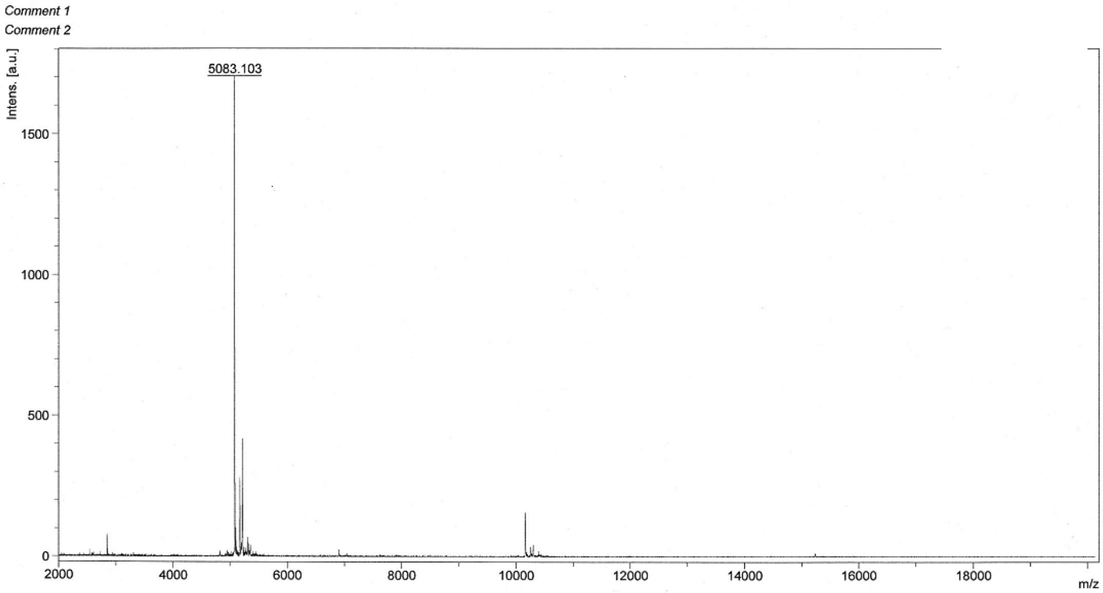

Figure S9. PNA3: K_3-CCTCT-AEEA-TCTCCCTTCTTT-K.

MALDI-TOF-MS (sinapinic acid): m/z calcd. 5082.51, observed 5083.10 [M(average)]$^+$.

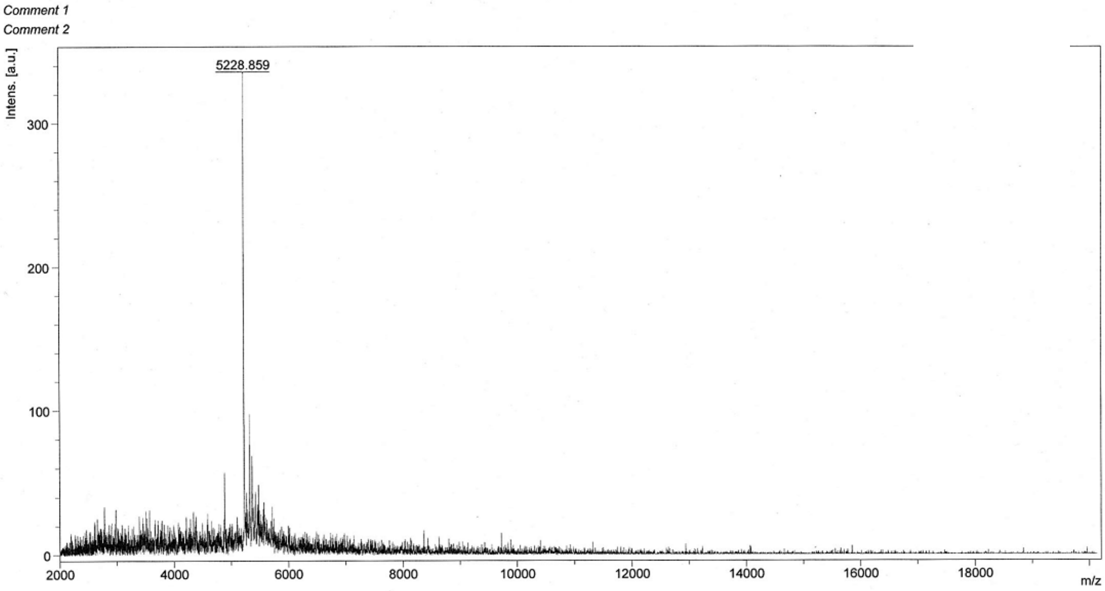

Figure S10. PNA4: K_3-CCTCT-$(AEEA)_2$-TCTCCCTTCTTT-K.

MALDI-TOF-MS (sinapinic acid): m/z calcd. 5227.7, observed 5228.86 [M(average)] $^+$.

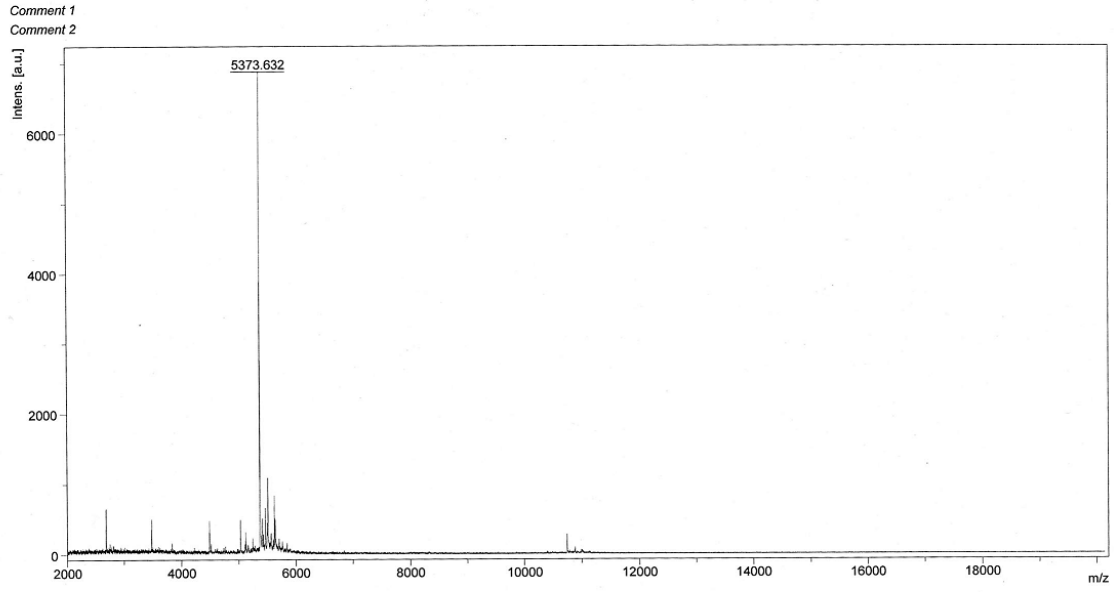

Figure S11. PNA**5**: K_3-CCTCT-$(AEEA)_3$-TCTCCCTTCTTT-K.

MALDI-TOF-MS (sinapinic acid): m/z calcd. 5372.8, observed 5373.63 [M(average)] $^+$.

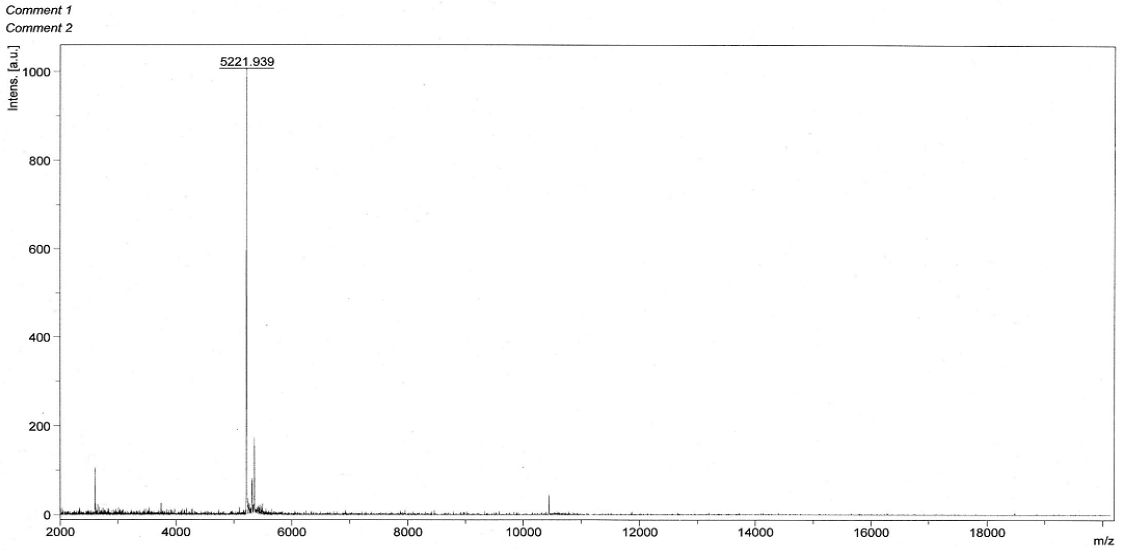

Figure S12. PNA**6**: K_3-CCTCT-AZO-TCTCCCTTCTTT-K.

MALDI-TOF-MS (sinapinic acid): m/z calcd. 5220.74, observed 5221.94 [M(average)]$^+$.

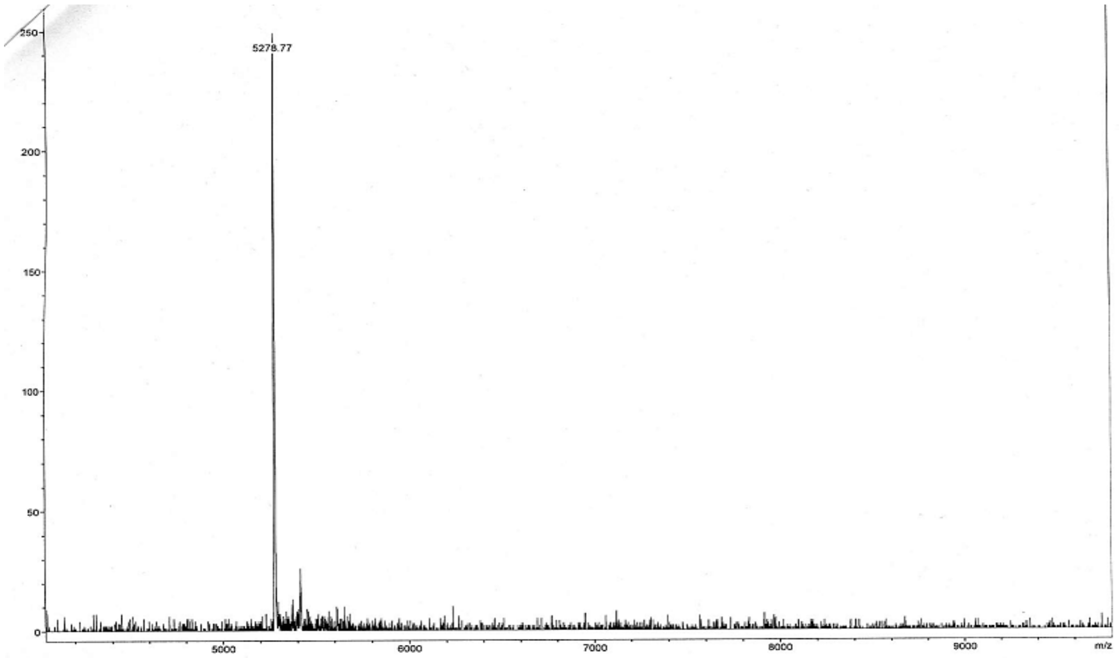

Figure S13. PNA7: K_3-CCTCT-C3-AZO-TCTCCCTTCTTT-K.

MALDI-TOF-MS (sinapinic acid): m/z calcd. 5277.81, observed 5278.77 [M(average)]$^+$.

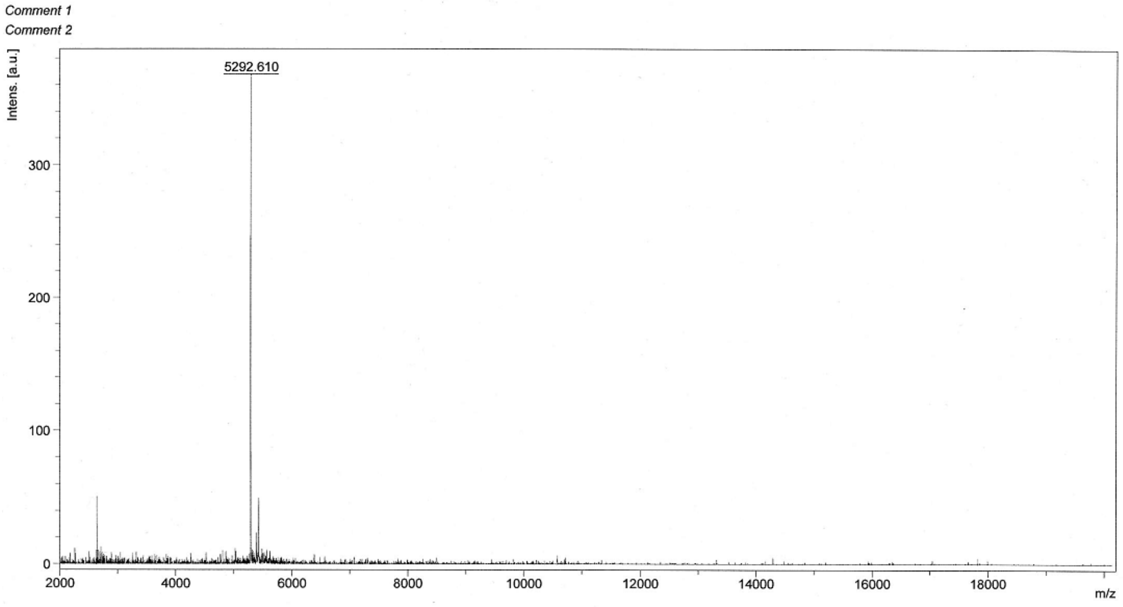

Figure S14. PNA8: K_3-CCTCT-C4-AZO-TCTCCCTTCTTT-K

MALDI-TOF-MS (sinapinic acid): m/z calcd. 5291.82, found 5292.61 [M(average)] $^{+}$.

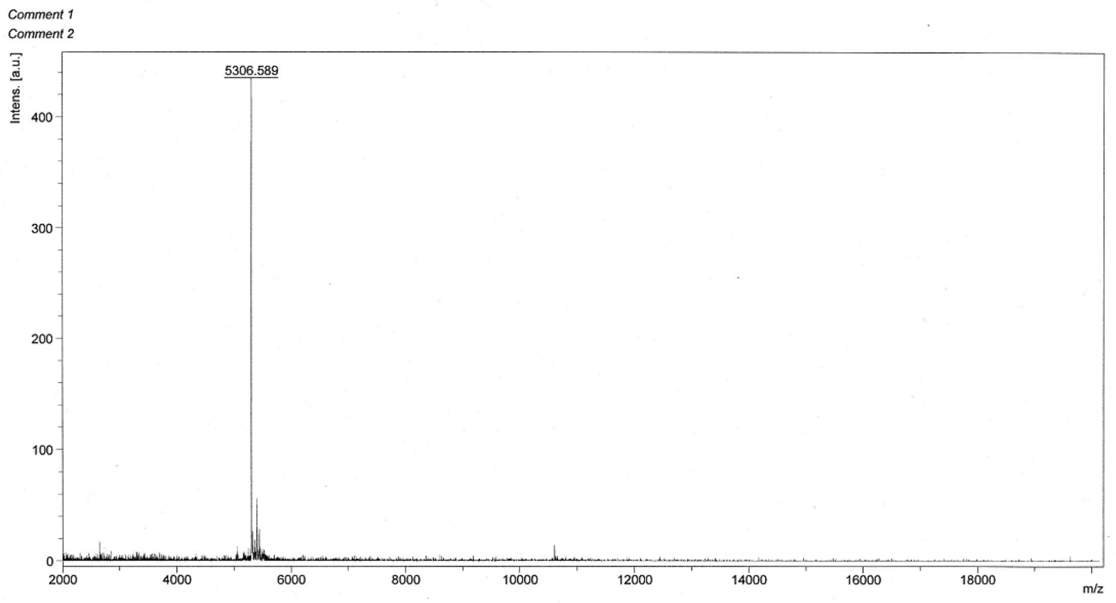

Figure S15. PNA**9**: K3-CCTCT-C5-AZO-TCTCCCTTCTTT-K

MALDI-TOF-MS (sinapinic acid): m/z calcd. 5305.86, observed 5306.59 [M(average)] $^{+}$.

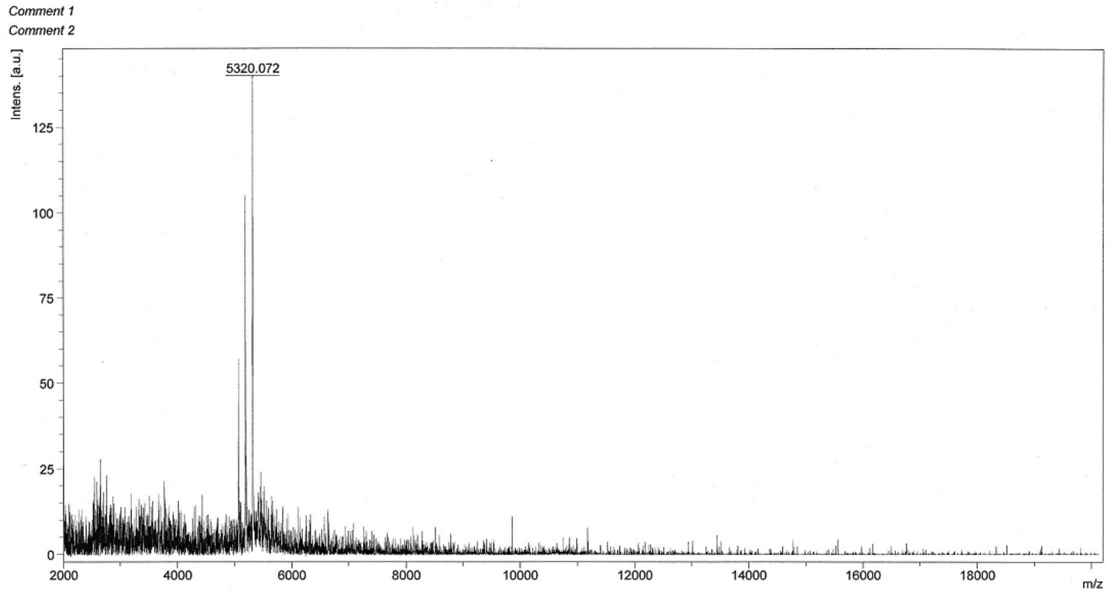

Figure S16. PNA**10**: K3-CCTCT-C6-AZO-TCTCCCTTCTTT-K.

MALDI-TOF-MS (sinapinic acid): m/z calcd. 5319.89, observed 5320.01 [M(average)]$^{+}$.

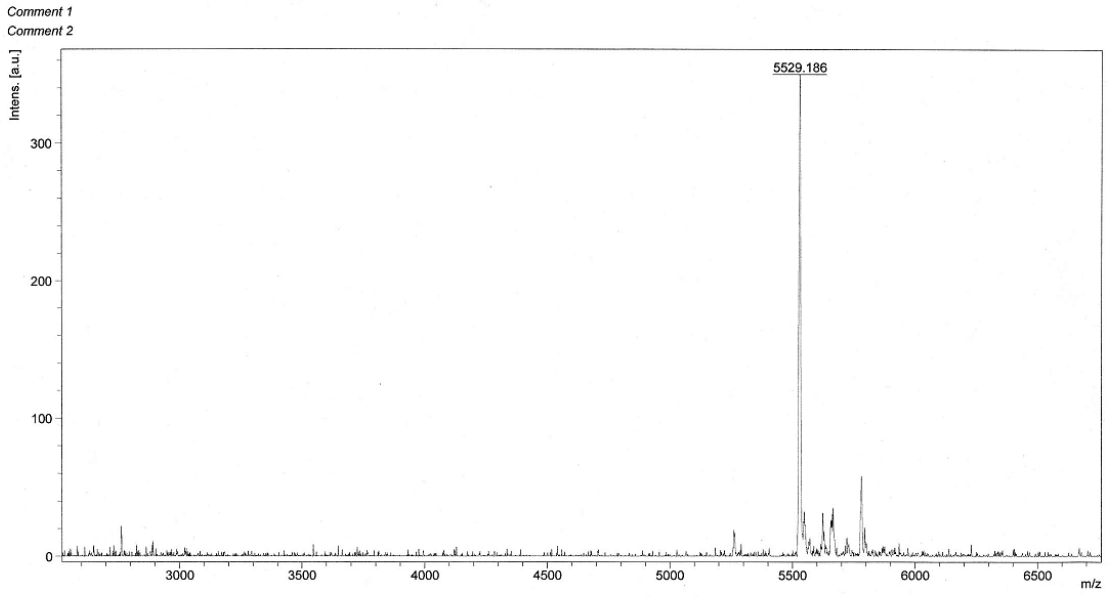

Figure S17. PNA**11**: K_3-CTTCCCTCT-C4-AZO-TCTCCCTTCTTT-K.

MALDI-TOF-MS (sinapinic acid): m/z calcd. 5528.17, observed 5529.19 [M(average)]$^{+}$.

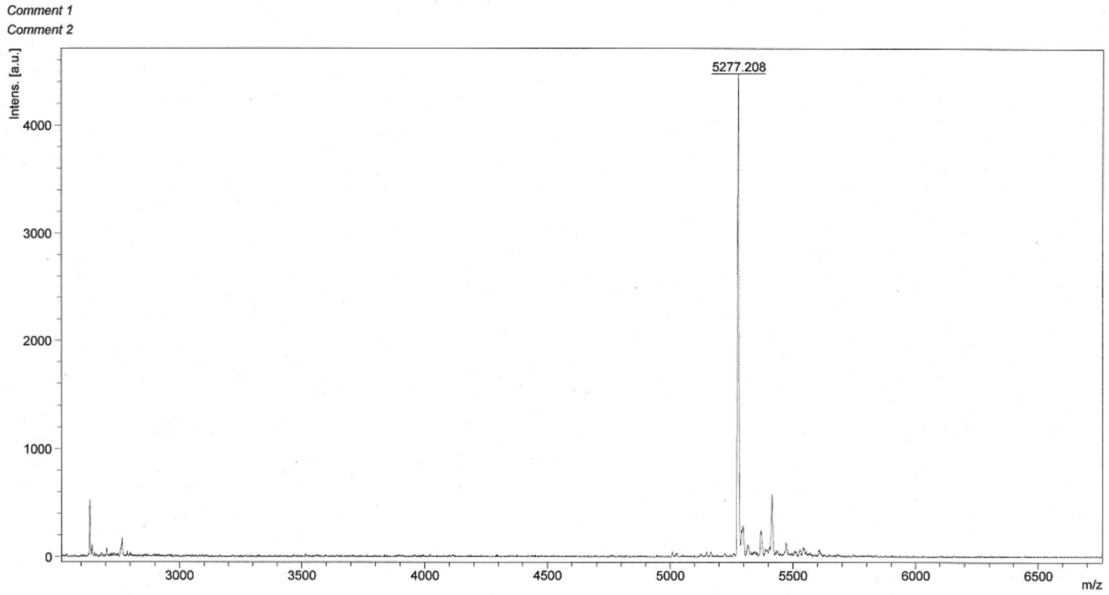

Figure S18. PNA**12**: K_3-TTCCCTCT-C4-AZO-TCTCCCTTCTTT-K.

MALDI-TOF-MS (sinapinic acid): m/z calcd. 5276.87, observed 5277.21 [M(average)]$^{+}$.

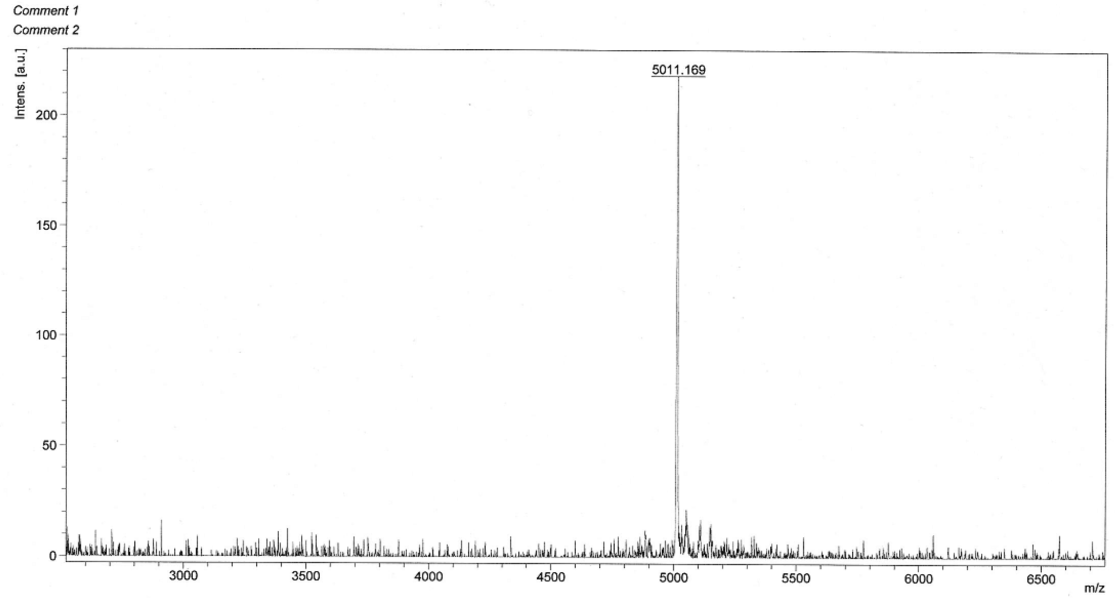

Figure S19. PNA**13**: K_3-TCCCTCT-C4-AZO-TCTCCCTTCTTT-K.

MALDI-TOF-MS (sinapinic acid): m/z calcd. 5010.62, observed 5011.17 [M(average)]$^{+}$.

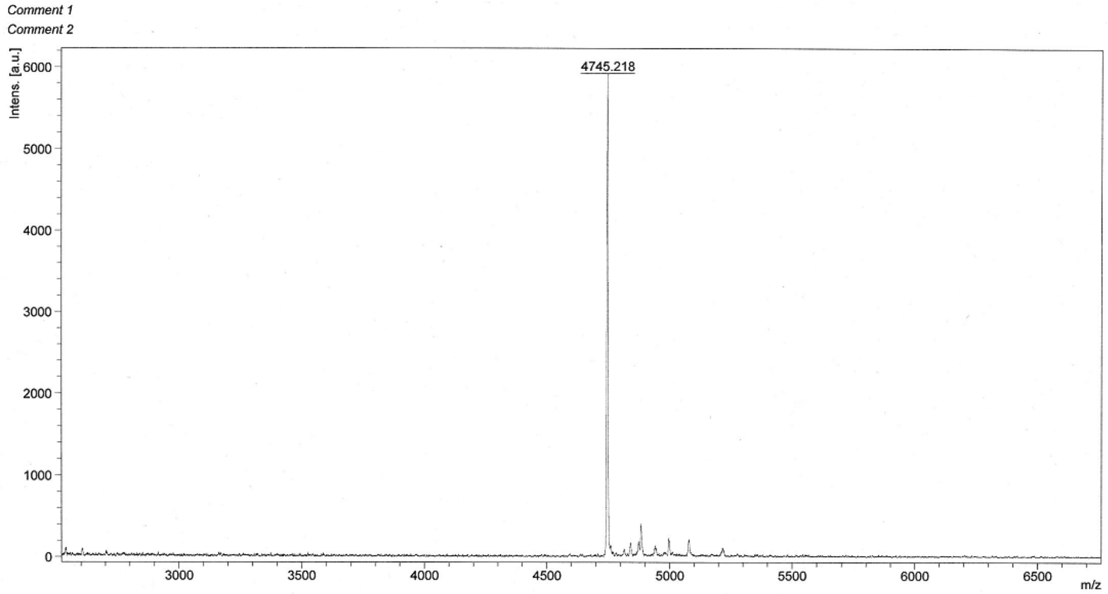

Figure S20. PNA**14:** K_3-CCCTCT-C4-AZO-TCTCCCTTCTTT-K.

MALDI-TOF-MS (sinapinic acid): m/z calcd. 4744.37, observed 4745.22 [M(average)]+.

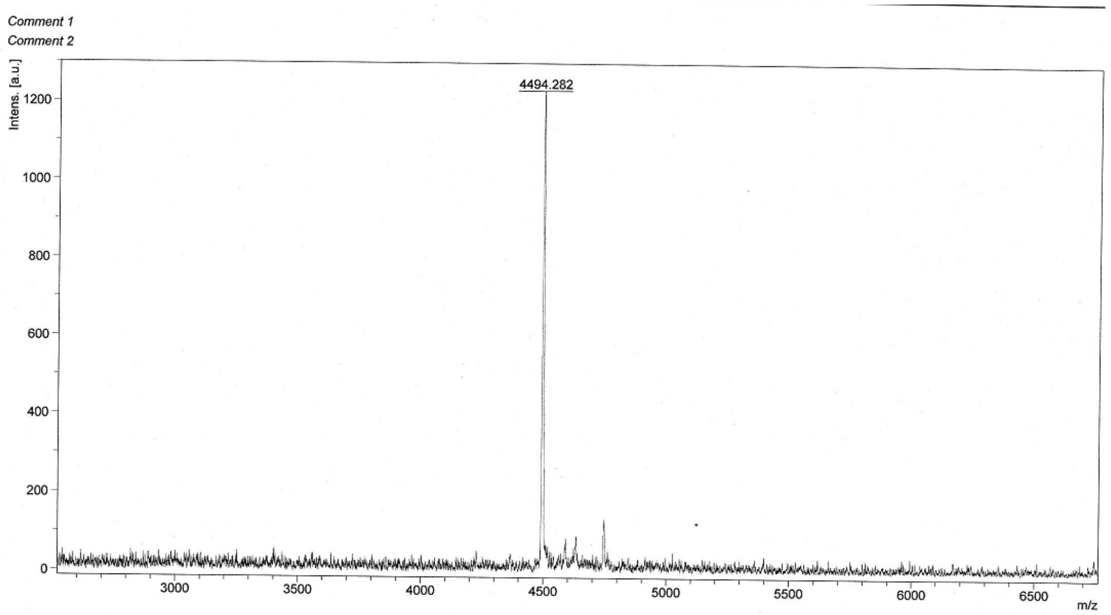

Figure S21. PNA**15**: K3-CCTCT-C4-AZO-TCTCCCTTCTTT-K.

MALDI-TOF-MS (sinapinic acid): m/z calcd. 4493.07, observed 4494.28 [M(average)] +.

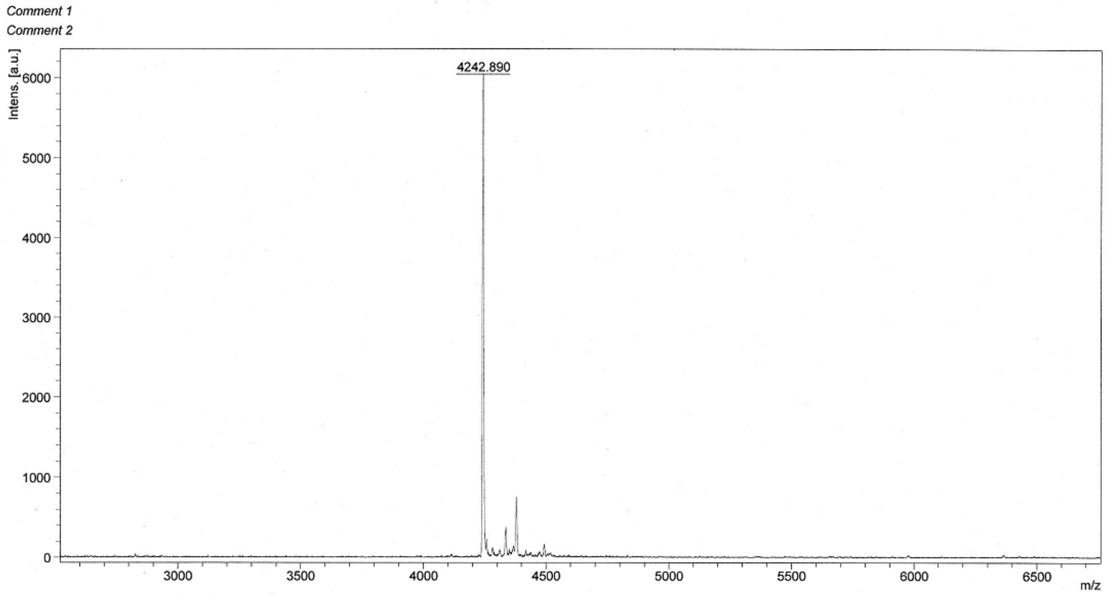

Figure S22. PNA**16**: K3-CTCT-C4-AZO-TCTCCCTTCTTT-K.

MALDI-TOF-MS (sinapinic acid): m/z calcd. 4241.77, observed 4242.89 [M(average)]$^{+}$.

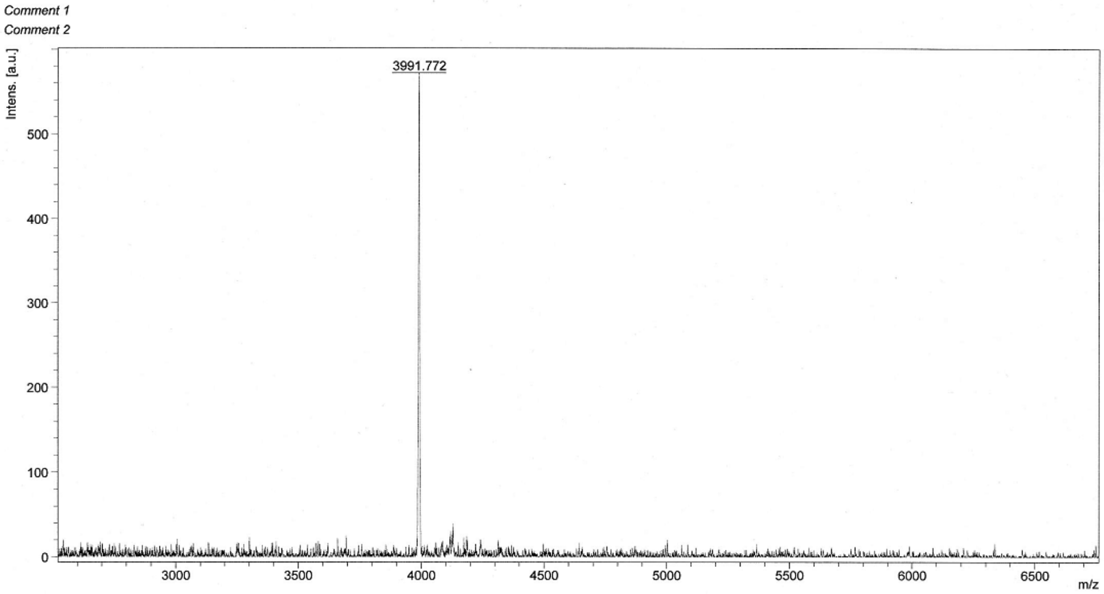

Figure S23. PNA**17**: K_3TCT-C4-AZO-TCTCCCTTCTTT-K.

MALDI-TOF-MS (sinapinic acid): m/z calcd. 3990.47, observed 3991.77 [M(average)]$^{+}$.

Table S1. Estimated multiply charged ion peaks of PNA**6**, DNA**1**, and their complexes. The highlighted peaks were identified in Figure S5A.

	Molecular weight
PNA **6**	5220.74
DNA 1	7982.50
Na^{+}	23.00

PNA/DNA/Na^{+}		PNA/PNA/DNA/Na^{+}		PNA/DNA/DNA/Na^{+}		DNA/Na^{+}		PNA/Na^{+}	
1	13227.24	1	18447.98	1	13250.24	1	8006.50	1	5244.74
2	6614.12	2	9224.49	2	6625.62	2	4003.75	2	2622.87
3	4409.75	3	6149.99	3	4417.41	3	2669.50	3	1748.91
4	3307.56	4	4612.75	4	3313.31	4	2002.38	4	1311.94
5	2646.25	5	3690.40	5	2650.85	5	1602.10	5	1049.75
6	2205.37	6	3075.50	6	2209.21	6	1335.25	6	874.96
7	1890.46	7	2636.28	7	1893.75	7	1144.64	7	750.11
8	1654.28	8	2306.87	8	1657.16	8	1001.69	8	656.47
9	1470.58	9	2050.66	9	1473.14	9	890.50	9	583.64
10	1323.62	10	1845.70	10	1325.92	10	801.55	10	525.37
11	1203.39	11	1678.00	11	1205.48	11	728.77	11	477.70
12	1103.19	12	1538.25	12	1105.10	12	668.13	12	437.98
13	1018.40	13	1420.00	13	1020.17	13	616.81	13	404.36
14	945.73	14	1318.64	14	947.37	14	572.82	14	375.55
15	882.75	15	1230.80	15	884.28	15	534.70	15	350.58
16	827.64	16	1153.94	16	829.08	16	501.34	16	328.73
17	779.01	17	1086.12	17	780.37	17	471.91	17	309.46
18	735.79	18	1025.83	18	737.07	18	445.75	18	292.32
19	697.12	19	971.89	19	698.33	19	422.34	19	276.99
20	662.31	20	923.35	20	663.46	20	401.28	20	263.19

Table S2. Estimated multiply charged ion peaks of PNA**8**, DNA**1**, and their complexes. The highlighted peaks were identified in Figure S5B.

	Molecular weight
PNA 8	5291.82
DNA 1	7982.50
Na^+	23.00

PNA/DNA/Na^+		PNA/PNA/DNA/Na^+		PNA/DNA/DNA/Na^+		DNA/Na^+		PNA/Na^+	
1	13298.32	1	18590.14	1	21280.82	1	8006.50	1	5315.82
2	6649.66	2	9295.57	2	10640.91	2	4003.75	2	2658.41
3	4433.44	3	6197.38	3	7094.27	3	2669.50	3	1772.61
4	3325.33	4	4648.29	4	5320.96	4	2002.38	4	1329.71
5	2660.46	5	3718.83	5	4256.96	5	1602.10	5	1063.96
6	2217.22	6	3099.19	6	3547.64	6	1335.25	6	886.80
7	1900.62	7	2656.59	7	3040.97	7	1144.64	7	760.26
8	1663.17	8	2324.64	8	2660.98	8	1001.69	8	665.35
9	1478.48	9	2066.46	9	2365.42	9	890.50	9	591.54
10	1330.73	10	1859.91	10	2128.98	10	801.55	10	532.48
11	1209.85	11	1690.92	11	1935.53	11	728.77	11	484.17
12	1109.11	12	1550.10	12	1774.32	12	668.13	12	443.90
13	1023.87	13	1430.93	13	1637.91	13	616.81	13	409.83
14	950.81	14	1328.80	14	1520.99	14	572.82	14	380.63
15	887.49	15	1240.28	15	1419.65	15	534.70	15	355.32
16	832.08	16	1162.82	16	1330.99	16	501.34	16	333.18
17	783.20	17	1094.48	17	1252.75	17	471.91	17	313.64
18	739.74	18	1033.73	18	1183.21	18	445.75	18	296.27
19	700.86	19	979.38	19	1120.99	19	422.34	19	280.73
20	665.87	20	930.46	20	1064.99	20	401.28	20	266.74

Table S3. Estimated multiply charged ion peaks of PNA**15**, DNA**1**, and their complexes. The highlighted peaks were identified in Figure S5C.

	Molecular weight
PNA 15	4507.11
DNA 1	7982.50
Na^+	23.00

PNA/DNA/Na^+		PNA/PNA/DNA/Na^+		PNA/DNA/DNA/Na^+		DNA/Na^+		PNA/Na^+	
1	12513.61	1	17020.72	1	20496.11	1	8006.50	1	4531.11
2	6257.31	2	8510.86	2	10248.56	2	4003.75	2	2266.06
3	4171.87	3	5674.24	3	6832.70	3	2669.50	3	1511.04
4	3129.15	4	4255.93	4	5124.78	4	2002.38	4	1133.53
5	2503.52	5	3404.94	5	4100.02	5	1602.10	5	907.02
6	2086.44	6	2837.62	6	3416.85	6	1335.25	6	756.02
7	1788.52	7	2432.39	7	2928.87	7	1144.64	7	648.16
8	1565.08	8	2128.47	8	2562.89	8	1001.69	8	567.26
9	1391.29	9	1892.08	9	2278.23	9	890.50	9	504.35
10	1252.26	10	1702.97	10	2050.51	10	801.55	10	454.01
11	1138.51	11	1548.25	11	1864.19	11	728.77	11	412.83
12	1043.72	12	1419.31	12	1708.93	12	668.13	12	378.51
13	963.51	13	1310.21	13	1577.55	13	616.81	13	349.47
14	894.76	14	1216.69	14	1464.94	14	572.82	14	324.58
15	835.17	15	1135.65	15	1367.34	15	534.70	15	303.01
16	783.04	16	1064.73	16	1281.94	16	501.34	16	284.13
17	737.04	17	1002.16	17	1206.59	17	471.91	17	267.48
18	696.15	18	946.54	18	1139.62	18	445.75	18	252.67
19	659.56	19	896.77	19	1079.69	19	422.34	19	239.43
20	626.63	20	851.99	20	1025.76	20	401.28	20	227.51

Table S4. Estimated multiply charged ion peaks of PNA**1**, DNA**1**, and their complexes. The highlighted peaks were identified in Figure S6A.

	Molecular weight
PNA 1	3266.44
DNA 1	7982.50
Na^+	23.00

PNA/DNA/Na^+		PNA/PNA/DNA/Na^+		PNA/DNA/DNA/Na^+		DNA/Na^+		PNA/Na^+	
1	11272.94	1	14539.38	1	19255.44	1	8006.50	1	3290.44
2	5636.97	2	7270.19	2	9628.22	2	4003.75	2	1645.72
3	3758.31	3	4847.13	3	6419.15	3	2669.50	3	1097.48
4	2818.99	4	3635.60	4	4814.61	4	2002.38	4	823.36
5	2255.39	5	2908.68	5	3851.89	5	1602.10	5	658.89
6	1879.66	6	2424.06	6	3210.07	6	1335.25	6	549.24
7	1611.28	7	2077.91	7	2751.63	7	1144.64	7	470.92
8	1409.99	8	1818.30	8	2407.81	8	1001.69	8	412.18
9	1253.44	9	1616.38	9	2140.38	9	890.50	9	366.49
10	1128.19	10	1454.84	10	1926.44	10	801.55	10	329.94
11	1025.72	11	1322.67	11	1751.40	11	728.77	11	300.04
12	940.33	12	1212.53	12	1605.54	12	668.13	12	275.12
13	868.07	13	1119.34	13	1482.11	13	616.81	13	254.03
14	806.14	14	1039.46	14	1376.32	14	572.82	14	235.96
15	752.46	15	970.23	15	1284.63	15	534.70	15	220.30
16	705.50	16	909.65	16	1204.40	16	501.34	16	206.59
17	664.06	17	856.20	17	1133.61	17	471.91	17	194.50
18	627.22	18	808.69	18	1070.69	18	445.75	18	183.75
19	594.26	19	766.18	19	1014.39	19	422.34	19	174.13
20	564.60	20	727.92	20	963.72	20	401.28	20	165.47

Table S5. Estimated multiply charged ion peaks of PNA**2**, DNA**1**, and their complexes. The highlighted peaks were identified in Figure S6B.

	Molecular weight
PNA 2	3549.80
DNA 1	7982.50
Na^+	23.00

PNA/DNA/Na^+		PNA/PNA/DNA/Na^+		PNA/DNA/DNA/Na^+		DNA/Na^+		PNA/Na^+	
1	11556.30	1	15106.10	1	19538.80	1	8006.50	1	3573.80
2	5778.65	2	7553.55	2	9769.90	2	4003.75	2	1787.40
3	3852.77	3	5036.03	3	6513.60	3	2669.50	3	1191.93
4	2889.83	4	3777.28	4	4885.45	4	2002.38	4	894.20
5	2312.06	5	3022.02	5	3908.56	5	1602.10	5	715.56
6	1926.88	6	2518.52	6	3257.30	6	1335.25	6	596.47
7	1651.76	7	2158.87	7	2792.11	7	1144.64	7	511.40
8	1445.41	8	1889.14	8	2443.23	8	1001.69	8	447.60
9	1284.92	9	1679.34	9	2171.87	9	890.50	9	397.98
10	1156.53	10	1511.51	10	1954.78	10	801.55	10	358.28
11	1051.48	11	1374.19	11	1777.16	11	728.77	11	325.80
12	963.94	12	1259.76	12	1629.15	12	668.13	12	298.73
13	889.87	13	1162.93	13	1503.91	13	616.81	13	275.83
14	826.38	14	1079.94	14	1396.56	14	572.82	14	256.20
15	771.35	15	1008.01	15	1303.52	15	534.70	15	239.19
16	723.21	16	945.07	16	1222.11	16	501.34	16	224.30
17	680.72	17	889.54	17	1150.28	17	471.91	17	211.16
18	642.96	18	840.17	18	1086.43	18	445.75	18	199.49
19	609.17	19	796.01	19	1029.31	19	422.34	19	189.04
20	578.77	20	756.26	20	977.89	20	401.28	20	179.64
